# The Transcription Factor DPB Confers Antiviral Defence Against Potato Virus X by Modulating MYB‐Dependent Signalling

**DOI:** 10.1111/mpp.70319

**Published:** 2026-07-12

**Authors:** Jingjing Shi, Yumei Zhao, Chunyan Qi, Yaoyao Jiang, Bin Yong, Lingyun Lei, Wenhao Wang, Peng Liu, Jiaqian Liu, Tianye Zhang, Jianping Chen, Jian Yang, Tianbo Liu, Kaili Zhong

**Affiliations:** ^1^ State Key Laboratory for Quality and Safety of Agro‐Products, Key Laboratory of Biotechnology in Plant Protection of MARA, Key Laboratory of Green Plant Protection of Zhejiang Province, Institute of Plant Virology Ningbo University Ningbo China; ^2^ Ministry of Agriculture Key Laboratory of Molecular Biology of Crop Pathogens and Insects, Institute of Biotechnology Zhejiang University Hangzhou China; ^3^ Luohe Academy of Agricultural Science Luohe China; ^4^ Tobacco Research Institute of Hunan Province Changsha Hunan China

## Abstract

As a transcriptional co‐factor of E2F, DP proteins are typically involved in the regulation of plant cell‐cycle‐related processes. However, whether DP proteins participate in transcriptional regulation associated with plant antiviral defence remains largely unclear. This study demonstrates that NbDPB, a DP family protein in *Nicotiana benthamiana*, positively regulates resistance to potato virus X (PVX) by functioning as a transcription factor. Knockout of *NbDPB* increased plant susceptibility to PVX, while its overexpression significantly suppressed viral accumulation. Furthermore, we identified that NbDPB binds to the promoter of *NbMYB* (a MYB transcription factor) and positively regulates its expression. Silencing *NbMYB* enhanced PVX infection, whereas exogenous application of jasmonic acid (JA) and salicylic acid (SA) partially rescued this phenotype. Hormone levels of JA and SA, as well as the expression levels of their marker genes, were significantly reduced in the silenced *NbMYB* plants. These findings suggest that the NbDPB‐NbMYB module mediates antiviral defence through the JA and SA signalling pathways. This study reveals a previously uncharacterized role of DP proteins in antiviral transcriptional regulation, indicating that they may play a crucial role in plant antiviral defence.

## Graphical abstract

This study identified that NbDPB confers resistance to potato virus X by directly activating the transcription factor NbMYB, which engages a hormone‐signalling network likely involving jasmonic acid and salicylic acid pathways.

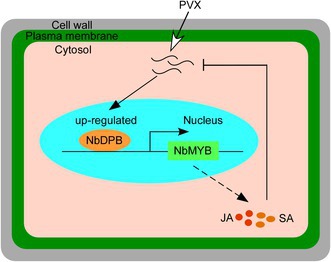

## 1 Introduction

Transcriptional reprogramming is a central feature of plant defence, serving as a molecular bridge between pathogen perception and physiological response. This reprogramming is primarily regulated by transcription factors (TFs), which act as critical hubs for signal integration. Notably, the functions of TFs extend beyond direct immune regulation to include the essential coordination of defence responses with developmental processes, such as cell cycle progression—a key interface for balancing growth and defence (Almagro et al. 2009; Ding et al. 2011; Huot et al. 2014). A paradigm for such coordination is the RB‐E2F pathway, a core transcriptional module that controls cell division and differentiation. In this pathway, E2F transcription factors typically form heterodimers with Dimerization Partner (DP) proteins to bind promoter regions and regulate the expression of cell cycle genes. While the E2F‐DP module is conserved, its specific functions and regulatory mechanisms can vary (Perrotta et al. 2021; Olson et al. 2010; Ramirez‐Parra et al. 2003; Li et al. 2021). In wheat, studies have identified and cloned the gene encoding a DP protein, revealing its conserved DP domain and widespread expression. Functional analyses indicate that wheat DP proteins enhance E2F‐DNA binding and stabilize the transcriptional complex (Ramírez‐Parra et al. 1999). DP proteins are primarily regarded as essential cofactors for E2F; however, their potential roles independent of E2F in plants, particularly in stress responses, remain largely unexplored and warrant further investigation. In contrast, the recognition and functional characteristics of DP proteins in plants have long remained unclear (Breeden 1996).

Among the numerous transcription factors involved in defence, the MYB family is one of the largest in plants, regulating diverse developmental processes and responses to environmental stimuli, such as cell fate determination and biotic and abiotic stresses (Stracke et al. 2001; Fernández‐Calvo et al. 2011; Li 2015; Amorim et al. 2017). Most plant MYB genes encode R2R3‐MYB proteins, which are believed to have evolved from an R1R2R3‐MYB ancestor, followed by gene family expansion (Kim, Gilmour, et al. 2020; Ding and Redkar 2018). This expanded family broadly regulates plant‐specific processes, ranging from metabolism and development to stress responses. Notably, several MYB transcription factors also play key roles in various defence mechanisms. For instance, AtMYB72 is required in the early signalling steps of rhizobacteria‐induced systemic resistance in *Arabidopsis* (Van der Ent et al. 2008). AtMYB96 serves as a molecular link integrating abscisic acid (ABA) and salicylic acid (SA) signals during pathogen‐induced expression of pathogenesis‐related (PR) genes in *Arabidopsis*, and AtMYB15 is essential for the induction of lignin as a component of the basal defence mechanism in the plant innate immune response (Seo and Park 2010; Chezem et al. 2017; Kim, Lam, et al. 2020). Other MYB transcription factors, including AtMYB30 and poplar MYB115, are also involved in plant pathogen responses (Vailleau et al. 2002; Wang et al. 2017). In addition to the canonical R2R3‐MYB family, members of the MYB‐related GOLDEN2‐LIKE (GLK) transcription factors have also emerged as important regulators of plant immunity. OsGLK1 acts as a key GLK transcription factor conferring rice resistance to rice black‐streaked dwarf virus (Li et al. 2022). AhGLK1b enhances resistance to both fungi and bacteria and activates disease‐related genes (Ali et al. 2020).

Understanding the function of these transcription factors often requires placing them within the context of phytohormone regulation. Plant hormones, including jasmonic acid (JA), SA and ABA, have long been implicated in responses to pathogens (Kodama and Kodama 1993; Zhao and Li 2021; Mengiste et al. 2003). R2R3‐MYB transcription factors are key mediators of defence responses downstream of these critical hormones. They not only respond to SA signals, as evidenced by the induction of PR genes and systemic resistance (Han and Kahmann 2019; Wang et al. 2022; Chen et al. 2017; Jung et al. 2008), but also integrate JA signalling pathways. For example, in rice, the JA‐responsive OsJMTF1 enhances resistance against bacterial blight by coordinating JA and auxin signalling (Uji et al. 2024), while OsMYB4P acts as a direct target of JAZ repressors to positively regulate JA‐mediated antiviral defence (Lu et al. 2025). MYB96 enhances plant disease resistance by mediating ABA signals that positively induce SA biosynthesis, demonstrating its role in responding to and amplifying SA‐mediated defence (Seo and Park 2010). In addition to R2R3‐MYB, R1‐related MYB also involved in hormone‐mediated disease resistance. For example, AtHRS1 mediates the plant response to nematodes by regulating JA‐dependent defence pathways (Wiśniewska et al. 2022). AtGLK1/GLK2 function redundantly to positively regulate resistance to cucumber mosaic virus by mediating antioxidant systems, defence gene expression and SA/JA signalling (Murmu et al. 2014). The role of relevant MYB transcription factors in the cross‐regulation of JA‐SA signalling has been clearly demonstrated in the literature, showing that the aforementioned MYB transcription factors are all involved in JA‐SA cross‐regulation. OsMYB4P functions primarily through JA signalling, and its antiviral activity can be synergistically enhanced by the SA pathway (Lu et al. 2025); the anti‐nematode function of AtHRS1 depends on the JA pathway, and SA can enhance its activation of JA defence genes via the NPR1 pathway (Wiśniewska et al. 2022).

Potato virus X (PVX) is a plant‐pathogenic virus belonging to the family *Alphaflexiviridae*. Its host plants are members of the *Solanaceae* family, such as potatoes and tomatoes (Kutnjak et al. 2014). PVX infection can cause various symptoms, including leaf crinkling and mild to severe mosaic patterns, and is primarily transmitted through mechanical means (Pourrahim et al. 2007; Seganti et al. 2004). Notably, there are no reports of fungal or insect vectors for PVX (Seganti et al. 2004). Additionally, PVX‐infected *Nicotiana occidentalis* P1 and *Nicotiana glutinosa* exhibit systemic and local necrotic lesions and mild mosaic symptoms, respectively (Balogun and Teraoka 2005). Research in *Nicotiana benthamiana* indicates that SA positively regulates resistance to PVX, highlighting its critical role in antiviral defence (Sánchez et al. 2010). In *N. benthamiana*, the JA signal regulated by COI1 controls the sesquiterpene phytoalexin synthesis genes *NbTPS1* and *NbEAH*, contributing to the basic antiviral defence against PVX (Li et al. 2015). StSWEET1g binds to viral capsid proteins by forming polymers, thereby disrupting the CP‐StHSP70 interaction and activating the JA pathway, which confers antiviral activity against PVX (Fang et al. 2025). This defensive function extends to MYB transcription factors beyond model plants; for example, in *Nicotiana* species, MYB1 is essential for *N* gene‐mediated resistance to tobacco mosaic virus (TMV) and contributes to the activation of *PR* gene expression (Liu et al. 2004; Yang and Klessig 1996). While MYBs are broadly implicated in plant disease resistance, their specific mechanisms of action and interactions with phytohormones such as SA during PVX infection remain unclear.

In the present study, we identified two DP proteins from *N. benthamiana*, and phylogenetic analysis revealed that they are highly conserved with DP proteins from *Arabidopsis* and rice. The *NbDPB* knockout mutants (*Cr‐nbdpb*) exhibited significantly increased host susceptibility to PVX infection, whereas overexpression of *NbDPB* enhanced host resistance. Integrated analysis of transcriptome and chromatin immunoprecipitation (ChIP)‐seq data identified a MYB transcription factor as a target gene of NbDPB. Silencing *NbMYB* increased PVX infection, whereas exogenous application of JA or SA restored PVX resistance in *NbMYB*‐silenced mutants.

## 2 Results

### 2.1 Identification and Analysis of DP in *N. benthamiana*, *Arabidopsis thaliana* and *Oryza sativa*

**FIGURE 1:**
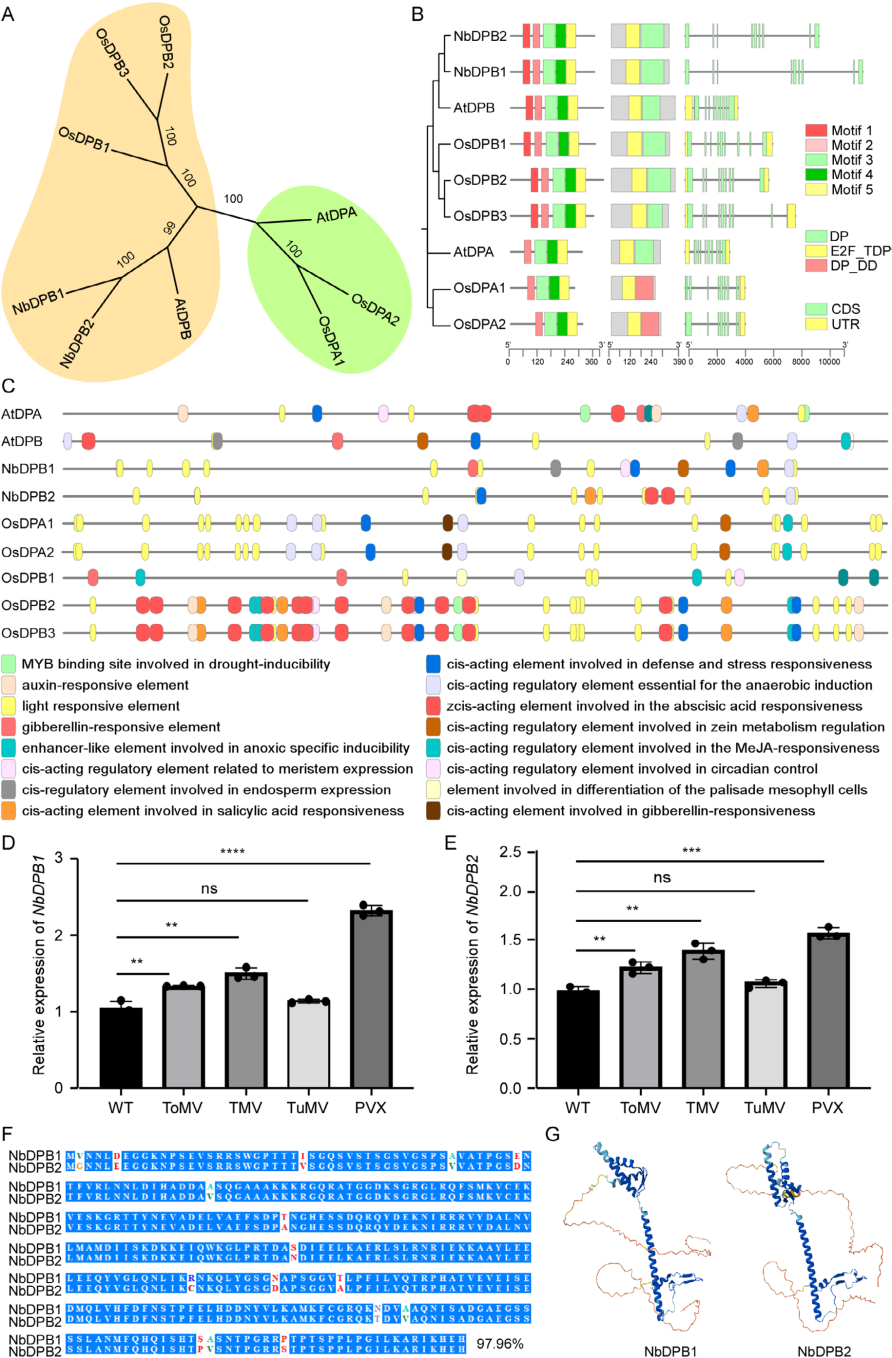
Whole‐genome analysis of the DP protein family and validation of associated *cis*‐acting elements. (A) Phylogenetic tree of DP proteins from *Arabidopsis thaliana* , *Nicotiana benthamiana* and *Oryza sativa* . The tree was constructed using the neighbour‐joining method in MEGA 11 software with 1000 bootstrap replicates. The DP proteins are divided into two subclasses, represented by distinct coloured backgrounds: DPA and DPB. (B) Conserved domain analysis, gene structure and conserved motifs of the DP protein family. Distribution of all motifs identified by MEME. Different coloured frames represent distinct protein motifs, each assigned a unique number. Each gene contains conserved domains, distinguished by different colours. Exon–intron structures of nine DP genes are shown. Exons, introns and untranslated regions are indicated by yellow boxes, grey lines and green boxes, respectively. (C) Names and positions of *cis*‐acting regulatory elements in the DP genes. (D, E) Relative expression levels of *NbDPBs* following viral infection (tomato mosaic virus [ToMV], tobacco mosaic virus [TMV], turnip mosaic virus [TuMV] and potato virus X [PVX]). WT refers to a plant that has been injected with infiltration buffer without virus. Three independent replicates were performed. Data are presented as mean ± SD (one‐way ANOVA, ns means no significant difference *p* > 0.05, **p < 0.01, ***p < 0.001, ****p < 0.0001). (F) Protein sequence alignment of NbDPB1 and NbDPB2 reveals 97.96% amino acid identity between the two proteins. (G) 3D structural prediction of NbDPB1 and NbDPB2 proteins using AlphaFold.

After conducting a genome‐wide search for DP homologues based on the AtDP genes in *A. thaliana* (Magyar et al. 2000), we identified two candidate DP genes in *N. benthamiana* and five candidate DP genes in *O. sativa* . To elucidate the evolutionary relationships and classify DPs in *A. thaliana* , *N. benthamiana* and *O. sativa* , we constructed a phylogenetic tree using two NbDPs, two AtDPs and five OsDPs. The DP family was classified into DPA and DPB subfamilies based on phylogenetic analysis (Figure 1A). Amino acid sequence analysis showed that the three members of the DPA subfamily ranged from 240 to 300 amino acids in length, while the six members of the DPB subfamily ranged from 300 to 390 amino acids. The six genes in the DPB subfamily contained five motifs (represented by Motifs 1–5), whereas three genes in the DPA subfamily contained only four motifs (represented by Motifs 2–5) (Figure 1B and Figure S1). The motif pattern is consistent with and helps highlight the distinction between the two subfamilies. Domain analysis of the proteins revealed that all members contain a DNA‐binding domain (E2F_TDP), a dimerization domain (DP or DP_DD) and a conserved MARKED box region. Although both DPA and DPB contain the conserved domains, differences in dimerization domain organization were found, and DPB is distinguished by a longer C‐terminal extension and specific amino acid insertions within the dimerization helix. Additionally, the exon‐intron distribution diagram revealed variation in gene organization among different DP family members. *NbDPB* lacks untranslated regions (UTRs), distinguishing it from the others (Figure 1B). Next, we conducted an analysis using the promoter region upstream of the gene and predicted the *cis*‐acting regulatory elements of NbDPs, AtDPs and OsDPs, identifying 16 distinct elements. The most abundant were the light‐responsive elements, followed by hormone‐responsive elements. Notably, we also identified defence‐ and stress‐responsive elements, which are particularly relevant to our study (Figure 1C). Accordingly, we investigated changes in the expression of *NbDPB*s under biotic stress by inoculating *N. benthamiana* with common infecting viruses: tomato mosaic virus (ToMV), turnip mosaic virus (TuMV), TMV and PVX. At 7 days post‐inoculation (dpi), leaves from equivalent positions were harvested for reverse transcription‐quantitative PCR (RT‐qPCR) analysis. The expression levels of *NbDPB1* and *NbDPB2* were significantly upregulated following ToMV, TMV and PVX infection, whereas TuMV infection had no detectable effect on their expression. Notably, PVX infection induced the most pronounced upregulation of *NbDPB1* and *NbDPB2* among all viral treatments tested (Figure 1D,E). Sequence alignment of NbDPB1 and NbDPB2 showed 97.96% amino acid identity (Figure 1F). Consistent with this, structural modelling demonstrated that the two proteins adopt analogous folding patterns, with conserved core domains and topological features (Figure 1G). Based on these findings, we selected NbDPB1 for subsequent experiments.

### 2.2 NbDPB Has No Effect on the Growth and Development of *N. benthamiana*

**FIGURE 2:**
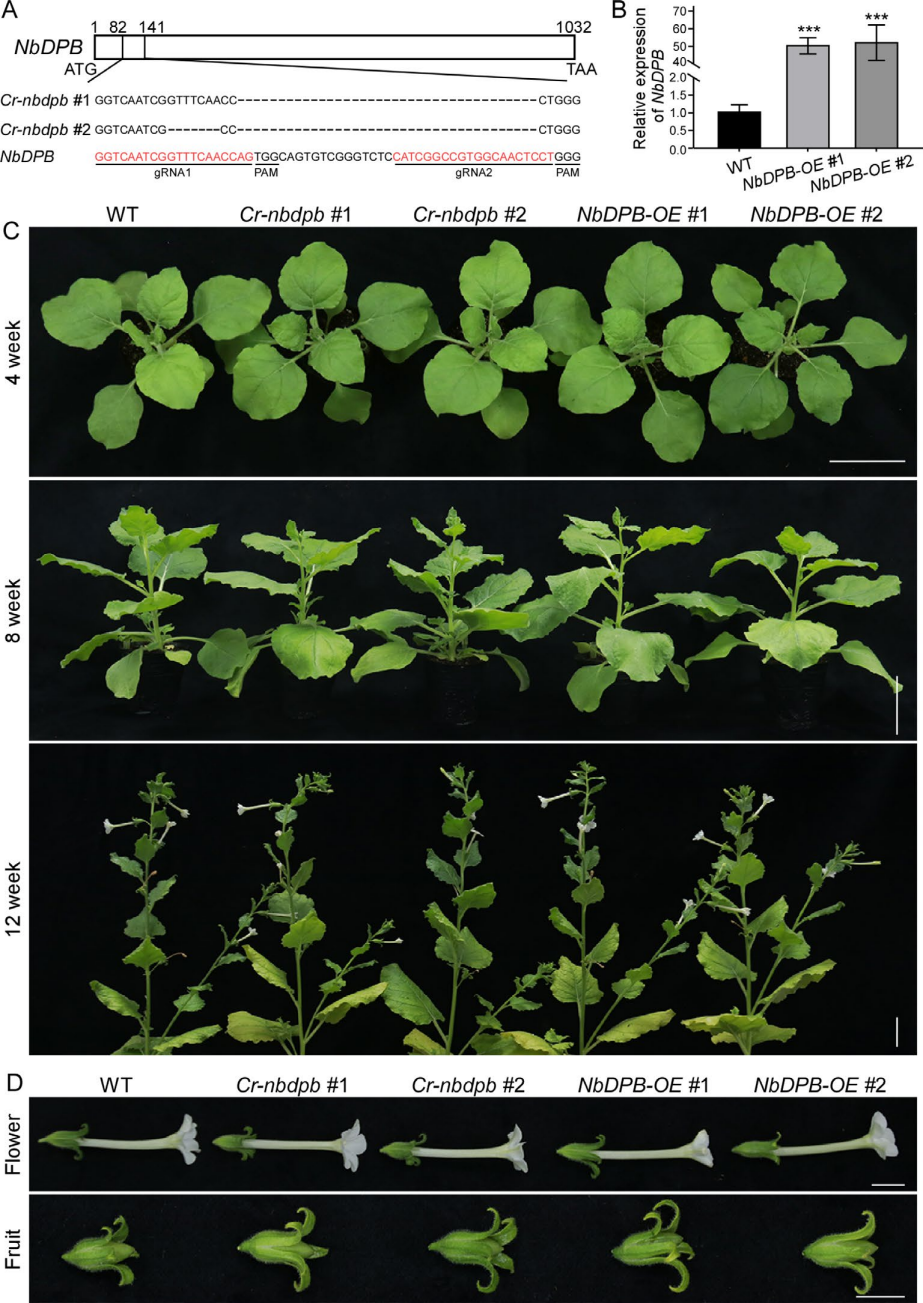
Growth and developmental phenotypes of CRISPR/Cas9‐edited *NbDPB* plants and *NbDPB* transgenic overexpression plants NbDPB‐edited lines. (A) Schematic representation of CRISPR/Cas9‐mediated knockdown of *NbDPB*. The PAM sequence (Protospacer Adjacent Motif) is the target DNA sequence to which Cas9 binds and cleaves. The red‐marked bases indicate the guide RNA (gRNA) sequence that recognizes the target genomic region. (B) The *NbDPB* overexpression vector driven by the CaMV 35S promoter and carrying a C‐terminal GFP tag was transgenic transformed into *Nicotiana benthamiana*. Two stable overexpression lines, *NbDPB‐OE*#1 and *NbDPB‐OE*#2 were verified by reverse transcription‐quantitative PCR analysis. WT, wild type. (C) Phenotypic comparison of *NbDPB*‐silenced mutants and overexpression mutants with WT plants at 4, 8 and 12 weeks. Bar = 10 cm. (D) Comparison of flower and fruit phenotypes between *NbDPB*‐silenced mutants, overexpression mutants, and WT plants. Bar = 1 cm.

To elucidate the functions of NbDPB in plant development in *N. benthamiana*, we used the CRISPR/Cas9‐mediated genome‐editing tool to knockout both *NbDPB1* and *NbDPB2*, and obtained two stable lines (*Cr*‐*nbdpb#1 and Cr‐nddpb#2*) (Figure 2A). Sequencing analysis confirmed successful editing of both *NbDPB1* and *NbDPB2* in the selected lines (Figure S2A,B). An *NbDPB* overexpression vector driven by the CaMV 35S promoter and carrying a C‐terminal GFP tag was generated. Two stable overexpression lines, *NbDPB‐OE#1* and *NbDPB‐OE#2*, were obtained via transgenic transformation. The overexpression of *NbDPB* was verified by RT‐qPCR (Figure 2B) and western blot analysis (Figure S2C). There were no significant differences in plant height or leaf size among *Cr‐nbdpb*, *NbDPB*‐*OE*, and wild‐type (WT) plants when observed at 4, 8 and 12 weeks (Figure 2C). Additionally, flowering and fruit setting were observed in *Cr‐nbdpb*, *NbDPB‐OE* and wild‐type (WT) plants. No obvious differences in plant growth or reproductive development were observed between *Cr‐nbdpb*, *NbDPB‐OE* and WT plants under the experimental conditions tested (Figure 2D). These observations suggest that alteration of *NbDPB* expression does not lead to obvious macroscopic developmental phenotypes in *N. benthamiana*, although potential functional redundancy or roles in cell‐cycle‐associated regulation cannot be excluded.

### 2.3 NbDPB Positively Regulates *N. benthamiana* Resistance to PVX

**FIGURE 3:**
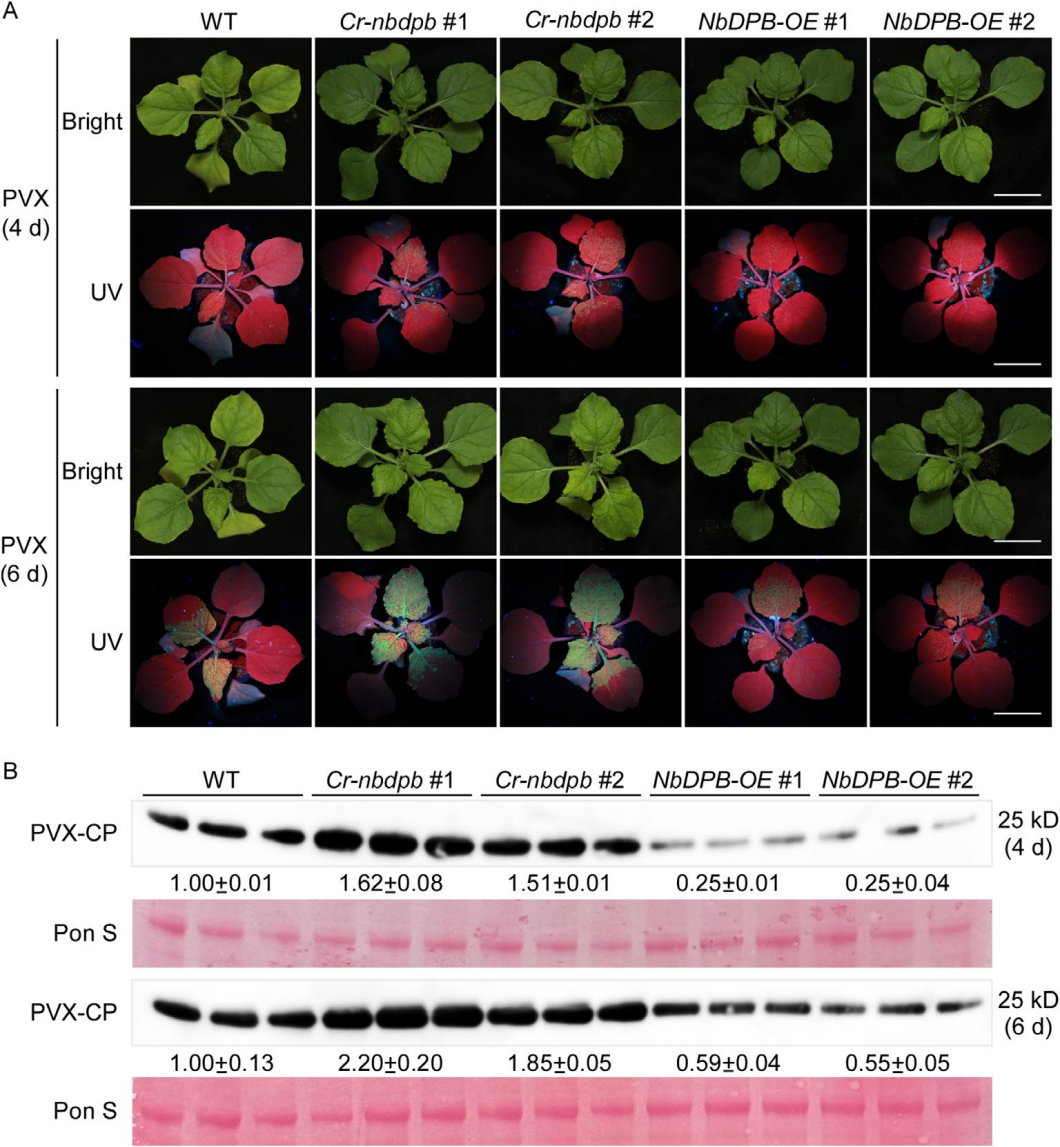
NbDPB positively regulates *Nicotiana benthamiana* resistance to potato virus X (PVX). (A) Phenotypes of *NbDPB‐*silenced mutants, *NbDPB*‐overexpression (OE) mutants and wild‐type (WT) plants after PVX‐GFP infection. Photographs were taken at 4 and 6 days post‐inoculation (dpi). The plants inoculated with PVX‐GFP showed systemic green fluorescence under UV illumination. Increased GFP fluorescence intensity indicates enhanced PVX accumulation. Bar = 5 cm. (B) Western blot detection of the protein content of PVX coat protein (CP) in WT, *NbDPB* knockout mutant, and *NbDPB* overexpression mutant plants. PVX was inoculated on *NbDPB* knockout mutant, *NbDPB* overexpression mutant, and WT plants, and the samples were taken at 4 and 6 dpi. The Ponceau S‐stained RuBisCO gel is used to show sample loadings. The numbers below the bands mean the intensity ratio of the three biological replicate bands that were calculated by ImageJ software. There were three biological replicates.

To investigate the role of NbDPB in PVX infection, GFP‐labelled PVX was inoculated into *Cr*‐*nbdpb*, *NbDPB‐OE* and WT plants via agroinfiltration. At 4 and 6 days post‐inoculation (dpi), plants inoculated with PVX‐GFP showed systemic green fluorescence under UV illumination, confirming successful viral replication and GFP expression. *Cr‐nbdpb* plants exhibited more intense green fluorescence signals, indicating enhanced PVX accumulation, together with more severe mosaic symptoms compared to WT plants. In contrast, *NbDPB‐OE* plants displayed weaker green fluorescence signals and milder mosaic symptoms than WT plants (Figure 3A). Furthermore, western blot analysis revealed that the accumulation of PVX coat protein (CP) was significantly higher in PVX‐inoculated *Cr‐nbdpb* plants than in WT plants and lower in *NbDPB‐OE* plants at both 4 and 6 dpi (Figure 3B). These results suggest that NbDPB positively regulates *N. benthamiana* resistance to PVX.

### 2.4 Genomic Identification of NbDPB Targets

**FIGURE 4:**
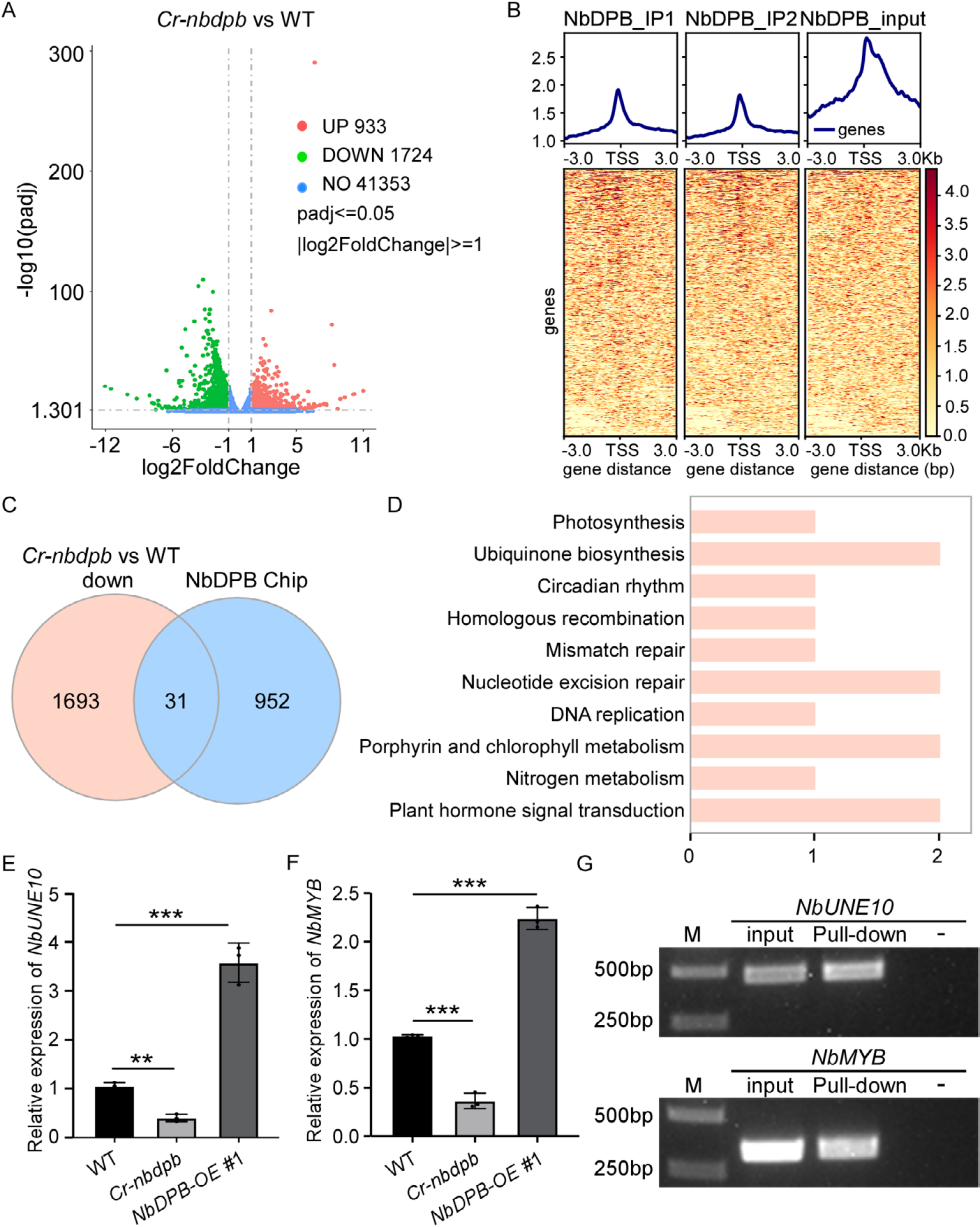
Identified genes potentially bound and regulated by NbDPB in antiviral responses. (A) Volcano plot showing the differentially expressed genes (DEGs) between *Cr‐nbdpb* and wild‐type (WT). The *x*‐axis indicates fold change in expression, and the *y*‐axis indicates the statistical significance. The size of each dot represents the significance of the *p*‐value. (B) Metagene plots and heatmaps showing NbDPB and enrichment at the NbDPB‐binding sites. TSS, transcription start site. Peaks aligned at the peak summit of NbDPB are plotted with 3‐kb upstream and downstream regions. The *y*‐axis of metagene plots indicates genes. In the profile plot, the vertical axis represents the average signal value (normalized read counts). (C) Venn diagram showing significant overlaps in the indicated gene sets. Significance of the overlaps was determined using a two‐sided Fisher's exact test. (D) KEGG functional statistics bar chart for 31 genes, excluding those without KEGG analysis. The *x*‐axis represents the number of genes, and the *y*‐axis represents KEGG functional categories. (E, F) The relative expression of *NbMYB* and *NbUNE10* in wild‐type (WT), *Cr‐nbdpb* and *NbDPB‐OE*. Data presented are the mean ± SD of three biological samples per treatment. Significant differences between treatments were determined using one‐way ANOVA, **p < 0.01, ***p < 0.001. (G) PCR detection of *NbMYB* and *NbUNE10*'s peaks in positive control group (input), experimental IP group (Pull‐down) and negative control (−). Water was used as the negative control.

To investigate whether NbDPB functions as a transcription factor regulating the expression of downstream genes, we performed transcriptome analysis on *Cr‐nbdpb* and WT plants. In the transcriptomic data, genes with a corrected *p‐*value ≤ 0.05 and an absolute fold change ≥ 2 were considered differentially expressed genes (DEGs). Differences between *Cr‐nbdpb* and WT were visualized using volcano plots, identifying a total of 1724 downregulated genes (Figure 4A and Table S1). To identify the potential DNA binding sites and target genes of NbDPB, we conducted ChIP‐Seq analysis using *NbDPB‐*GFP. Metagene plots and heatmaps showed that NbDPB is predominantly enriched at the transcription start sites of genes (Figure 4B). Consequently, 1024 NbDPB‐binding peaks (*p* ≤ 0.05), corresponding to 983 genes, were identified (Table S2). Intersection analysis of these two gene sets revealed 31 overlapping genes (Figure 4C and Table S3). To clarify whether the 31 downstream genes that bound and are regulated by NbDPB are associated with E2F, we analysed all known motifs of binding peaks for the 31 overlapping genes. These binding peaks include domains such as bZIP, AP2, EREBP and TCP; among them, only one gene contains an E2F‐binding motif (Table S4). To determine the functions of these 31 genes, we performed KEGG pathway analysis and found that two genes (*NbMYB*, Niben261Chr02g0981002 and *NbUNE10*, Niben261Chr08g0185006) are involved in plant hormone signal transduction (Figure 4D). *NbMYB* belongs to the GLK subfamily and is highly homologous to genes such as *HHO*/*HRS1* (Figure S4A). AtHRS1 regulates nematode resistance through a JA‐dependent pathway (Wiśniewska et al. 2022). AhGLK1b activates SA synthesis and disease‐resistance genes, enhancing resistance to fungi and bacteria (Ali et al. 2020). AtGLK1/2 mediates resistance to saprophytic fungi via SA signalling (Murmu et al. 2014). *NbUNE10* belongs to subfamily VII of the bHLH transcription factor family and is highly homologous to PIL/PIF (Figure S4B). PIL/PIF possess conserved and critical functions in plant immunity and interactions with pathogens (Oh et al. 2009). For example, PIF downregulates the JA signalling pathway reducing plant resistance to *Botrytis cinerea* (Xiang et al. 2020). Rice PIL15 forms a transcriptional complex with WRKY36 and downregulates plant resistance to sheath blight (Wang et al. 2025). Accordingly, *NbMYB* and *NbUNE10* may act as target genes of NbDPB participating in biotic stress or hormone signalling. The expression levels of these two genes were downregulated in *Cr‐nbdpb* plants but upregulated in *NbDPB‐OE* plants compared to WT, supporting a positive regulatory effect of NbDPB on their expression (Figure 4E,F). To further validate NbDPB binding to these genes, we performed ChIP‐PCR experiments. The results showed amplification of both genes in the immunoprecipitation (IP) and input groups, which is consistent with the ChIP‐Seq findings (Figure 4G).

### 2.5 NbDPB Is a Transcriptional Activator of *NbMYB*

**FIGURE 5:**
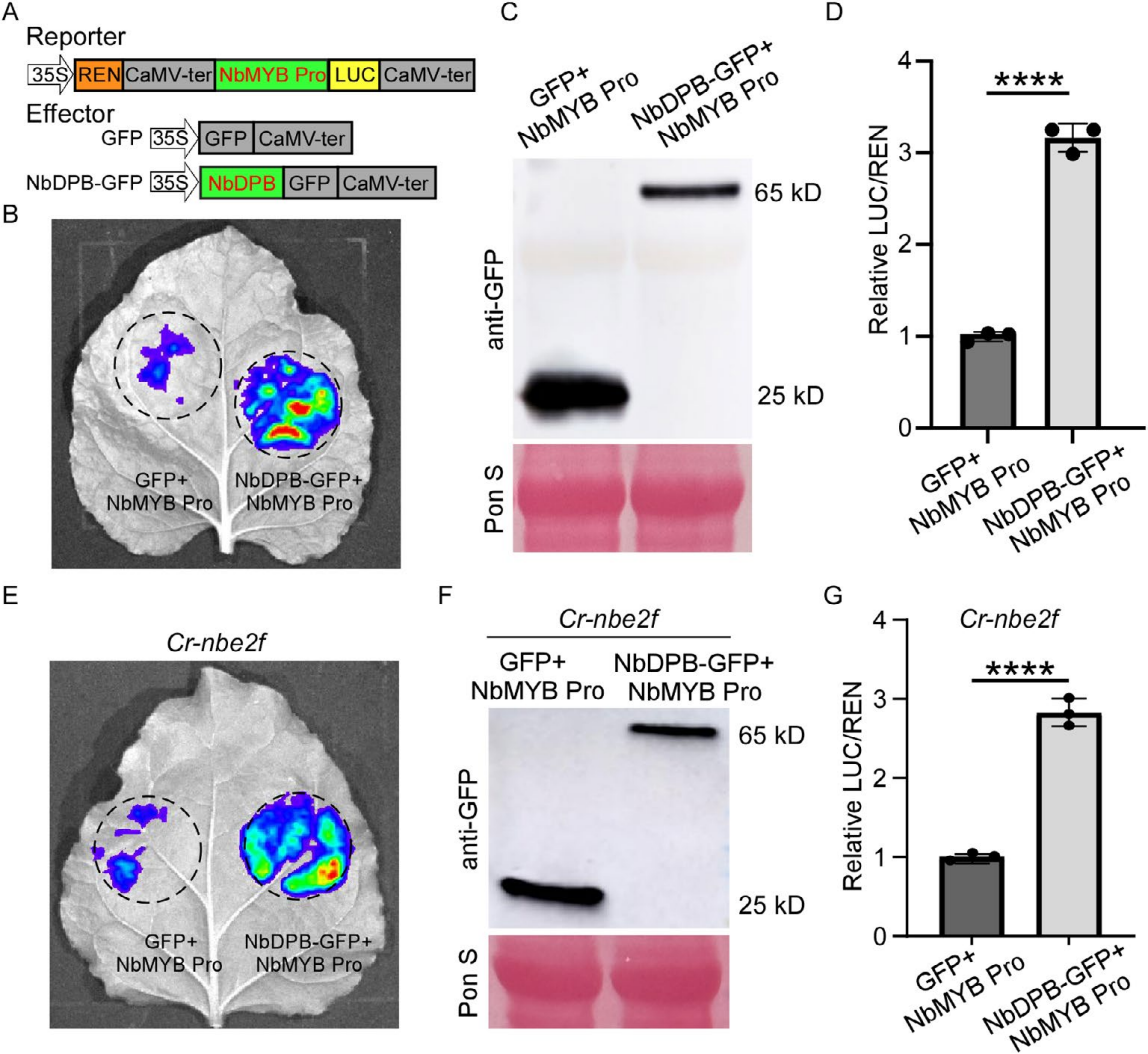
NbDPB is a transcriptional activator of *NbMYB*. (A) Schematic diagrams of the effectors and reporters (NbMYB Pro) used in the dual‐luciferase experiments. REN, renilla luciferase, an internal control; LUC, firefly luciferase. NbDPB fused with GFP tag driven by the CaMV 35S promoter (NbDPB‐GFP) and free GFP driven by CaMV 35S promoter (GFP) were the effectors. (B) Photograph of *Nicotiana benthamiana* leaves that were co‐infiltrated with each reporter and each effector measured at 48 h post‐infiltration (hpi); the sample with the GFP effector was used as the negative control. (C) The accumulation of GFP in the NbMYB Pro/GFP and NbMYB Pro/NbDPB‐GFP transiently expressed *N. benthamiana* leaves by western blot analysis. GFP was used as a negative control. (D) Relative luciferase activities were analysed by the LUC/REN ratio. The GFP effector was used as a negative control. Error bars represent SD; values are means ± SD (*n* = 3 biologically independent replicates per genotype). Significant difference was analysed using Student's *t*‐test, ****p < 0.0001. (E) Photograph of *Cr‐nbe2f* leaves that were co‐infiltrated with each reporter and each effector measured at 48 hpi; the sample with the GFP effector was used as the negative control. (F) The accumulation of GFP in the NbMYB Pro/GFP and NbMYB Pro/NbDPB‐GFP transiently expressed *Cr‐nbe2f* leaves by western blot analysis. GFP was used as a negative control. (G) Relative luciferase activities were analysed by the LUC/REN ratio. The GFP effector was used as a negative control. Error bars represent SD; values are means ± SD (*n* = 3 biologically independent replicates per genotype). Significant difference was analysed using Student's *t*‐test, ****p < 0.0001.

As a transcription factor, we primarily focused on identifying NbDPB‐binding genes with binding sites located in promoter regions. Therefore, we examined the annotations of the peaks from the two selected genes and found that the *NbMYB* peak was located in the promoter region, while the *NbUNE10* peak was in the distal intergenic region. Consequently, we selected *NbMYB* for further investigation. To validate the binding of NbDPB to the promoter region of *NbMYB*, we performed a dual‐luciferase transient transcriptional activity assay. The *NbMYB* promoter was cloned into a dual‐luciferase reporter vector as the reporter, NbDPB fused with GFP was expressed as the effector, and a 35S promoter GFP vector was used as the control (Figure 5A). Two days after injection into *N. benthamiana*, in vivo imaging results showed that NbDPB enhanced the expression level of *NbMYB* (Figure 5B). The expression of GFP and NbDPB‐GFP at the injection site was confirmed by western blot (Figure 5C). We further measured dual luciferase activity, and the results confirmed the promoting effect of NbDPB on *NbMYB* expression (Figure 5D). These results indicate that NbDPB acts as a transcriptional activator of *NbMYB*.

To determine whether NbDPB's positive regulation of *NbMYB* described here is independent of E2F, we used the CRISPR/Cas9‐mediated genome‐editing tool to knockout *NbE2F* and obtained one stable line (*Cr*‐*nbe2f*). Sequencing analysis confirmed successful editing of *NbE2F* in the selected line (Figure S5A–C). Then, we performed a dual‐luciferase transient transcriptional activity assay in the *Cr‐nbe2f* line. Two days after injection into *Cr‐nbe2f* leaves, in vivo imaging results showed that NbDPB enhanced the expression level of *NbMYB* in the absence of *NbE2F* (Figure 5E). The expression of GFP and NbDPB‐GFP at the injection site was confirmed by western blot (Figure 5F). Further dual luciferase activity results confirmed the promoting effect of NbDPB on *NbMYB* expression in the absence of *NbE2F* (Figure 5G). The results indicate that NbDPB acts as a transcriptional activator of *NbMYB*, and this action is independent of *NbE2F*.

### 2.6 NbMYB Positively Regulates *N. benthamiana* Resistance to PVX

**FIGURE 6:**
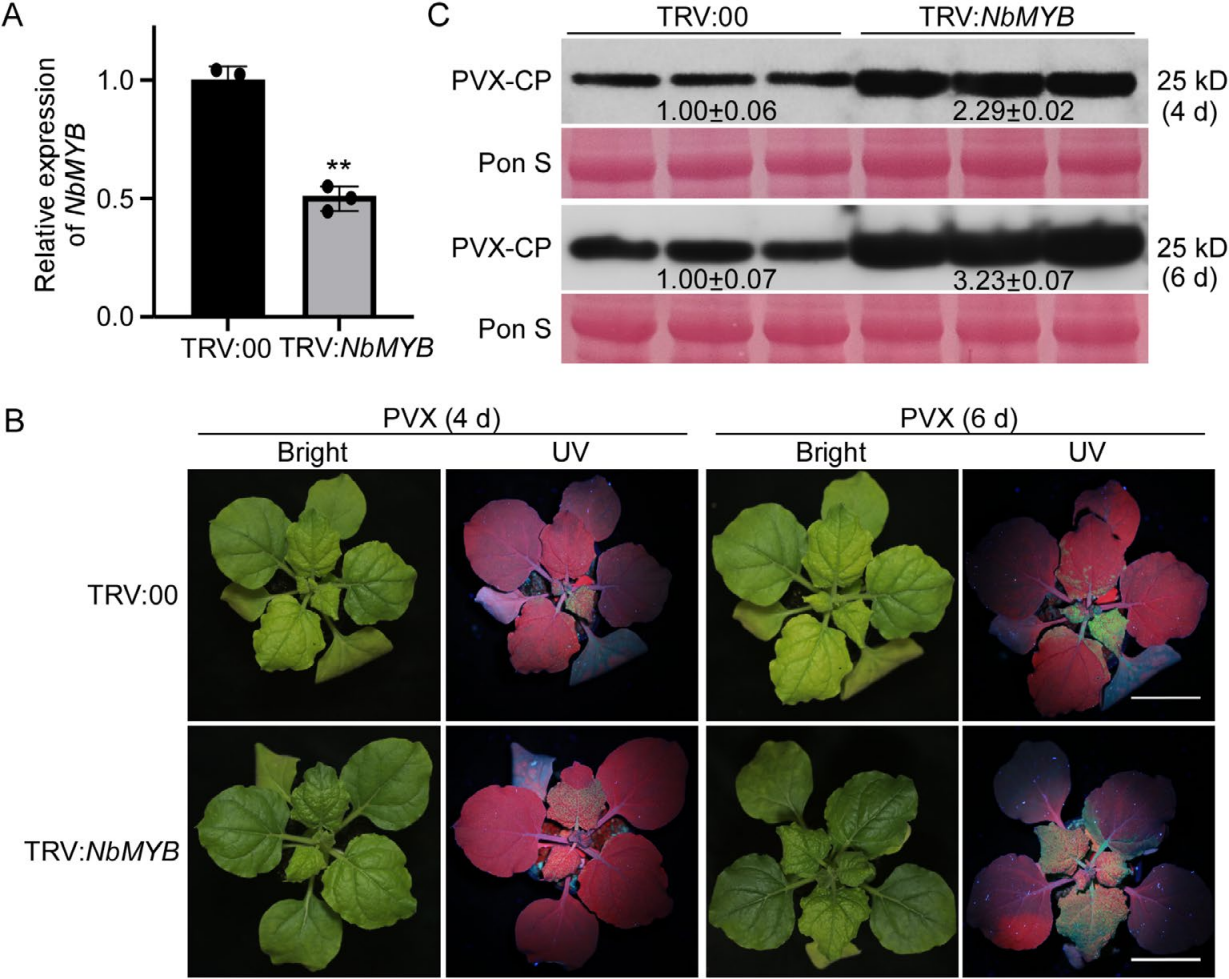
Silencing of *NbMYB* expression promotes potato virus X (PVX) infection. (A) Relative expression levels of *NbMYB* in the TRV:00‐ and TRV:*NbMYB*‐inoculated *Nicotiana benthamiana* plants determined through reverse transcription‐quantitative PCR. Data presented are the mean ± SD of three biological samples per treatment. Significant differences between treatments were determined using Student's *t*‐test, **p < 0.01. (B) Systemic mosaic symptoms on the PVX‐inoculated TRV:00 and TRV:*NbMYB* plants. Photographs were taken at 4 and 6 days post‐inoculation (dpi), the plants inoculated with PVX‐GFP showed systemic green fluorescence under UV illumination. Bar = 5 cm. (C) Western blot assay for PVX coat protein (CP) accumulation in PVX‐inoculated TRV:00 and TRV:*NbMYB* plant leaves. The protein content in the different samples was then determined by Ponceau S staining. The numbers below the bands mean the intensity ratio of the three biological replicate bands that were calculated by ImageJ software. There were three biological replicates.

To investigate the role of NbMYB in PVX infection, we silenced *NbMYB* expression in *N. benthamiana* plants using tobacco rattle virus (TRV)‐based virus‐induced gene silencing (VIGS) technology. At 7 dpi, RT‐qPCR results confirmed successful silencing of *NbMYB* in the treated plants (Figure 6A). These plants were then re‐inoculated with PVX fused with GFP. At 4 and 6 dpi, plants inoculated with PVX‐GFP showed systemic green fluorescence under UV illumination. *NbMYB*‐silenced plants (TRV:*NbMYB*) exhibited more intense green fluorescence and more severe mosaic symptoms compared to control plants (TRV:00) (Figure 6B). Furthermore, western blot analysis showed that the accumulation of PVX coat protein (CP) was significantly higher in PVX‐inoculated TRV:*NbMYB* plants than in TRV:00 plants at both 4 and 6 dpi (Figure 6C).

### 2.7 NbMYB Mediates the JA and SA Pathways Involved in PVX Infection

**FIGURE 7:**
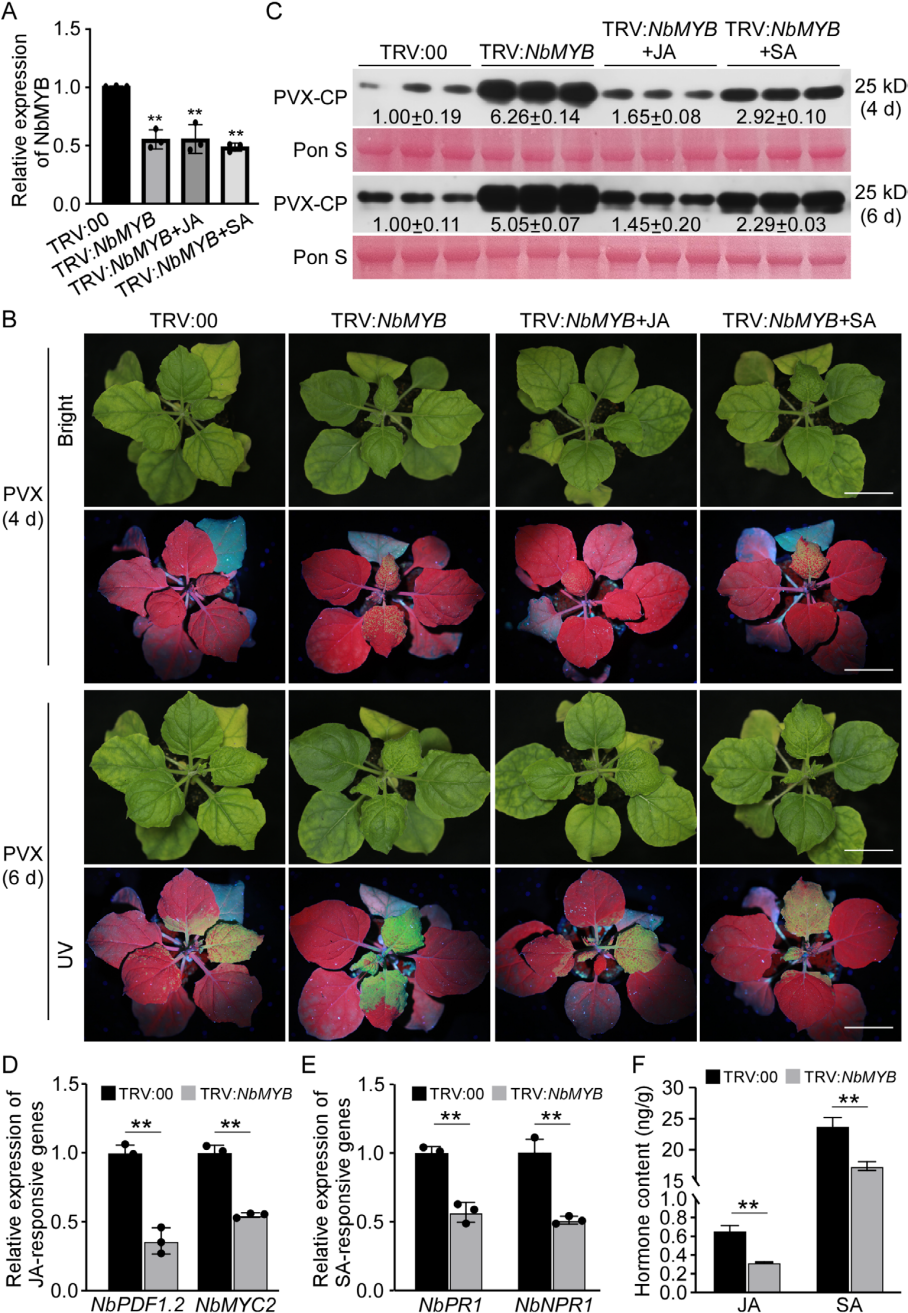
Jasmonic acid (JA) and salicylic acid (SA) reversed the silencing of *NbMYB*‐mediated enhancement of potato virus X (PVX) infection. (A) Relative expression levels of *NbMYB* in the TRV:00, TRV:*NbMYB*, TRV:*NbMYB*+JA and TRV:*NbMYB*+SA plants was determined by reverse transcription‐quantitative PCR. Data presented are the mean ± SD of three biological samples per treatment. Significant differences between treatments were determined using one‐way ANOVA, **p < 0.01. (B) Exogenous JA or SA treatment partially rescued the enhanced PVX susceptibility phenotype caused by *NbMYB* silencing. Representative systemic mosaic symptoms on the PVX‐inoculated TRV:00, TRV:*NbMYB*, TRV:*NbMYB*+JA and TRV:*NbMYB*+SA plants. Photographs were taken at 4 and 6 days post‐inoculation (dpi), the plants inoculated with PVX‐GFP showed systemic green fluorescence under UV illumination. Bar = 5 cm. (C) Western blot assay for PVX coat protein (CP) accumulation in PVX‐inoculated TRV:00, TRV:*NbMYB*, TRV:*NbMYB*+JA and TRV:*NbMYB*+SA plant leaves. The leaves' protein was taken at 4 and 6 dpi with PVX. The protein content in the different samples was then determined by Ponceau S staining. The numbers below the bands mean the intensity ratio of the three biological replicate bands that were calculated by ImageJ software. There were three biological replicates. (D, E) Relative expression levels of JA‐responsive marker genes (*NbPDF1.2* and *NbMYC2*) and SA‐responsive marker genes (*NbPR1* and *NbNPR1*) in TRV:*NbMYB* and TRV:00 *Nicotiana benthamiana* plants. Data presented are the mean ± SD of three biological samples per treatment. Significant differences between treatments were determined using two‐way ANOVA, **p < 0.01. (F) Endogenous JA and SA levels were measured in TRV:00 and TRV:*NbMYB*. Data presented are the mean ± SD of three biological samples per treatment. Significant differences between treatments were determined using two‐way ANOVA, **p < 0.01.

NbMYB belongs to the GLK subfamily within the R1‐related MYB family. Numerous reports have linked this subfamily to hormone‐mediated disease resistance. For example, AtHRS1 mediates plant response to nematodes by regulating JA‐dependent defence pathways (Wiśniewska et al. 2022). AtGLK1/2 enhance resistance to *B. cinerea* in an SA‐signal‐dependent manner (Murmu et al. 2014). AhGLK1 activates the key enzyme involved in chloroplast SA biosynthesis and PR10, enhancing resistance to bacteria and fungi, as well as tolerance to abiotic stress (Ali et al. 2020). Therefore, we hypothesized that NbMYB might also regulate the SA and JA signalling pathways. To test this hypothesis, we first silenced *NbMYB* in *N. benthamiana* plants using TRV‐based VIGS. At 7 dpi, RT‐qPCR results confirmed successful silencing of *NbMYB* in nine *NbMYB*‐silenced plants (TRV:*NbMYB*), which were divided into three groups (Figure 7A). At 4 and 6 dpi, plants inoculated with PVX‐GFP showed systemic green fluorescence under UV illumination, confirming successful viral replication and GFP expression. the TRV:*NbMYB* plants exhibited stronger green fluorescence signals, indicating enhanced PVX accumulation, together with more severe mosaic symptoms compared to controls (TRV:00). However, *NbMYB*‐silenced plants treated with JA and SA (TRV:*NbMYB*+JA and TRV:*NbMYB*+SA) showed reduced green fluorescence and milder mosaic symptoms, comparable to the control group (Figure 7B). Western blot analysis indicated that TRV:*NbMYB* plants experienced the most severe PVX infection relative to controls. Following treatment with SA and JA, PVX infection was partially alleviated, with JA demonstrating a greater degree of recovery than SA (Figure 7C). In addition, exogenous application of SA or JA to TRV:00 plants also affected PVX accumulation. However, the effect was far less pronounced than that observed in TRV:*NbMYB* plants (Figure S6A,B). To investigate the potential involvement of NbMYB in modulating hormone‐mediated defence responses, we examined the basal expression levels of core JA‐ and SA‐responsive marker genes in *NbMYB*‐silenced and control plants. Three *NbMYB*‐silenced plants were confirmed by RT‐qPCR results (Figure S6C). The results showed that silencing of *NbMYB* significantly reduced the expression levels of JA‐responsive genes (*NbPDF1.2* and *NbMYC2*) and SA‐responsive genes (*NbPR1* and *NbNPR1*) in comparison to TRV:00 (Figure 7D,E). We also measured the endogenous levels of JA and SA in TRV:00 and TRV:*NbMYB* plants. The results showed that the concentrations of both JA and SA were significantly reduced in the TRV:*NbMYB* plants (Figure 7F). These results indicate that NbMYB positively modulates the basal expression of key genes in both SA and JA signalling pathways.

## 3 Discussion

The DP protein family has been systematically identified and characterized in *Arabidopsis*; however, the family members and functions remain poorly understood in the widely used model plant *N. benthamiana*. In this study, we conducted a genome‐wide analysis of the DP gene family in three model species— *A. thaliana* , *O. sativa* and *N. benthamiana*. Nine DP genes were identified and grouped into two subfamilies, DPA and DPB. Phylogenetic analysis showed that DP proteins clustered into two conserved subfamilies across different plant species. Consistent with this phylogenetic clustering, motif composition and gene structural architecture analyses further revealed a generally conserved organization within the DP family, although some variations in motif composition and domain arrangement were observed among different members and species: DPB proteins contained conserved motifs together with canonical DP‐ and E2F‐associated domains, while several DPA proteins displayed additional domain features. *Cis*‐element analysis of the promoter regions of two *N. benthamiana* DPB genes revealed the presence of defence‐ and stress‐related elements. Guided by this observation, we examined their expression following infection with four different plant viruses and found that *NbDPB1* was most strongly upregulated under PVX infection (Figure 1). Furthermore, PVX inoculation revealed clear phenotypic differences: knockout of *NbDPB* resulted in earlier and more severe viral symptoms, whereas overexpression of *NbDPB* led to attenuated symptoms and reduced viral accumulation. These results indicate that NbDPB negatively regulates PVX infection and contributes to antiviral defence (Figure 3).

DP proteins, as transcriptional coactivators, typically form heterodimers with E2F to promote downstream gene expression, thereby regulating key cellular processes in plants, including mitosis, DNA damage response and repair, cell differentiation and programmed cell death (Bracken et al. 2004; Cam et al. 2004; DeGregori and Johnson 2006; Dimova and Dyson 2005; Ren et al. 2002). However, knockout or overexpression of *NbDPB* in *N. benthamiana* did not result in obvious differences in major developmental traits such as plant height, leaf morphology, flowering or seed setting (Figure 2). These observations indicate that alteration of *NbDPB* expression does not lead to obvious macroscopic developmental phenotypes, although potential functional redundancy or roles in cell‐cycle‐associated regulation cannot be excluded. Nevertheless, GO and KEGG analyses of transcriptomic (Figure S3A,B) and ChIP‐Seq (Figure S3C,D) revealed significant enrichment of genes and pathways associated with cell cycle regulation, DNA replication, and cell division, supporting the conserved involvement of NbDPB in canonical E2F‐DP‐associated functions. Therefore, the absence of visible developmental defects is more likely attributable to functional redundancy or compensatory regulatory mechanisms rather than a lack of participation in cell‐cycle regulation. Similar functional redundancy has also been reported for E2F family members in *Arabidopsis*, where individual mutations often produce only minor developmental phenotypes, whereas combinatorial mutants exhibit more pronounced developmental defects (Gombos et al. 2023).

Transcription factors are key regulators of plant immunity. For example, ERFs activate defence responses by binding to the GCC box (Nakano et al. 2006); tobacco MYB1 is induced by TMV and bacterial pathogens and activates PR gene expression (Yang and Klessig 1996); and numerous WRKY family members are pathogen‐responsive and play prominent roles in disease resistance (Ishiguro and Nakamura 1994). Here, NbDPB bound to 31 candidate genes that were also downregulated in *Cr‐nbdpb* compared to the WT. To clarify whether these 31 downstream genes are associated with E2F, we analysed all known motifs of binding peaks for 31 overlapping genes. These binding peaks include domains such as bZIP, AP2, EREBP and TCP; among them, only one gene contained an E2F‐binding motif (Table S4). This indicates that DPB may have non‐classical functions independent of E2F. It is also possible that E2F‐DPB heterodimers bind to promoter regions lacking the conserved E2F‐binding sequence, as demonstrated in animal studies (Rabinovich et al. 2008). Previous studies have primarily focused on DPB as an E2F‐associated transcriptional cofactor, whereas its potential involvement in antiviral transcriptional regulation has remained largely unexplored. Here, we identified a defence‐related gene *NbMYB*, in which a prominent binding peak was found in its promoter region (Figure 4). Based on these data, we hypothesized that NbDPB may regulate defence responses by activating *NbMYB* transcription. The dual‐luciferase assay showed that NbDPB significantly enhanced *NbMYB* activity relative to the control, demonstrating that NbDPB positively regulates *NbMYB*. Previous studies have implicated E2Fs in the regulation of defence responses (Ascencio‐Ibáñez et al. 2008; Wang et al. 2014). To determine whether NbDPB's positive regulation of *NbMYB* described here is independent of E2F, we performed the dual‐luciferase assay in *NbE2F* knockout (*Cr‐nbe2f*) plant leaves. The results showed that NbDPB enhanced the expression level of *NbMYB* in *Cr‐nbe2f* (Figure 5). Our findings indicate that NbDPB activates *NbMYB* independent of NbE2F, providing a mechanistic link to hormone‐mediated defence pathways.

The MYB family, particularly the R2R3‐MYB subfamily, is one of the largest transcription factor families in plants and serves as a key regulator of defence signalling, including JA and SA responses. For example, the JA‐responsive MYB transcription factor JMTF1 enhances rice resistance against bacterial blight by amplifying JA‐mediated defence signalling and upregulating key defence genes (Uji et al. 2024). MdMYB73 functions as a positive regulator that enhances resistance to *Botryosphaeria dothidea* in apple through the upregulation of SA‐mediated defence responses (Gu et al. 2021). To further clarify the evolutionary characteristics of NbMYB, we conducted a phylogenetic analysis. We found that this gene belongs to the GARP subfamily within the R1‐MYB family. In addition, the R1‐MYB family has also been reported to confer resistance to plant pathogens. For example, AtHRS1 mediates the plant's response to nematodes by regulating JA‐dependent defence pathways (Wiśniewska et al. 2022); the *Arabidopsis* GLK1/2 enhance resistance to the saprophytic fungus *B. cinerea* in an SA‐signal‐dependent manner (Murmu et al. 2014). Additionally, SA and JA have been shown to enhance PVX resistance in *N. benthamiana* (Sánchez et al. 2010; Fang et al. 2025; Li et al. 2015). In this study, we found that *NbMYB* silencing significantly repressed the accumulation of endogenous JA and SA, along with the transcript levels of their marker genes. Consistent with these changes, silencing *NbMYB* increased PVX susceptibility, and this phenotype could be partially rescued by exogenous JA and SA treatment (Figures 6 and 7). Our results suggest a model in which NbDPB, upon PVX challenge, activates NbMYB, which may in turn potentiate JA and SA signalling or regulate JA‐ and SA‐responsive defence genes, thereby establishing an antiviral state. Thus, NbMYB likely functions as a positive regulator of PVX resistance, potentially through amplifying JA‐ and SA‐mediated defence responses.

**FIGURE 8:**
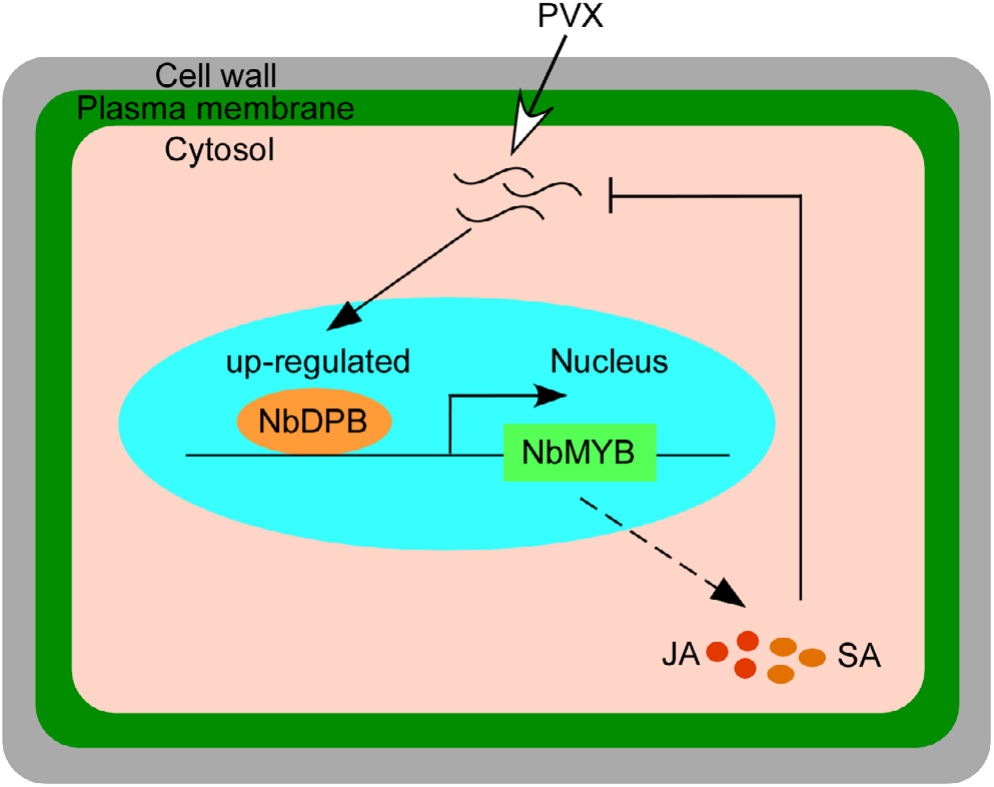
NbDPB confers antiviral defence against potato virus X (PVX) by modulating MYB‐dependent jasmonic acid (JA) and salicylic acid (SA) signalling pathways. Upon PVX infection, the expression of *NbDPB* is upregulated. Furthermore, NbDPB binds to the promoter of *NbMYB*, which mediates antiviral defence through both JA and SA pathways.

In summary, this study identified NbDPB in *N. benthamiana* and uncovered an additional role beyond its canonical E2F‐associated functions in antiviral immunity. NbDPB1 confers resistance to PVX by directly activating the downstream MYB transcription factor NbMYB, which appears to function within a hormone‐signalling network likely involving JA and SA (Figure 8). Whereas previous studies have focused on DPB as an E2F co‐activator in species such as *Arabidopsis* and wheat, this work highlights an independent transcriptional regulatory function for DPB in *N. benthamiana*, elucidating its molecular role during PVX infection and expanding our understanding of the functional diversity of the DP protein family. Nonetheless, several questions remain unresolved, such as identifying additional NbDPB target genes, exploring post‐transcriptional mechanisms mediated by NbDPB during infection and determining how this regulatory module influences viral replication, movement and host defence processes. Further research is needed to elucidate how NbMYB suppresses PVX infection and mediates the JA and SA hormone pathways, as well as the molecular mechanisms by which these pathways regulate the host's defence against viral infection.

## 4 Experimental Procedures

### 4.1 Identification of DP Genes in *N. benthamiana* and *O. sativa*

To identify potential DP genes in *N. benthamiana* (*NbDPs*) and *O. sativa* (*OsDPs*), the *A. thaliana* *AtDPA* (AT5G02470) and *AtDPB* (AT5G03415) were used to search against a hidden Markov model (HMM) (Magyar et al. 2000). TBtools software (v. 2.136) was employed to search the *N. benthamiana* database (*Nicotiana_benthamiana* V261) available on the Sol Genomics Network (https://solgenomics.net/, accessed 17 September 2025). Additional DP genes from rice were identified using the same approach. The *O. sativa* database (GCF_034140825.1_ASM3414082v1) was also downloaded from NCBI ( *Oryza sativa* Japonica Group genome assembly AGIS1.0—NCBI—NLM, accessed 17 September 2025). Members of the *NbDP* and *OsDP* gene families were verified using Pfam (https://www.ebi.ac.uk/interpro/search/sequence/, accessed 19 September 2025), and the NbDP protein sequences were also obtained.

### 4.2 Phylogenetic Analysis

Three datasets comprising two identified NbDP protein sequences, two DP protein sequences from *A. thaliana* (AtDPs), and five DP protein sequences from *O. sativa* (OsDPs) were used for phylogenetic analysis. Multiple sequence alignments were performed using MEGA 11 software (v. 11.0.13) with the MUSCLE algorithm. A phylogenetic tree was constructed using the neighbour‐joining method, and bootstrap analysis with 1000 replicates was conducted to assess the reliability of the results (Wu et al. 2024).

### 4.3 Gene Structural Domain, Gene Structure and Motif Analysis of NbDPs, OsDPs and AtDPs

The protein sequences of NbDPs, OsDPs and AtDPs were submitted to the NCBI Batch CD‐Search tool (https://www.ncbi.nlm.nih.gov/Structure/bwrpsb/bwrpsb.cgi, accessed on 23 September 2025). TBtools software (v. 2.136) was used to obtain and visualize gene structural domain data, including gene structure analysis via the TBtools Gene Structure View. Motif prediction for NbDPs, OsDPs and AtDPs was performed using the MEME Suite (https://memesuite.org/meme/tools/meme, accessed on 23 September 2025), an online analysis tool.

### 4.4 *Cis*‐Acting Regulatory Elements Analysis

The promoter regions of the *NbDP*, *OsDP* and *AtDP* genes, spanning 2 kb upstream of the transcription start site, were obtained from the *N. benthamiana*, *O. sativa* and *A. thaliana* databases. These DNA sequences were analysed to identify *cis*‐acting regulatory elements using the PlantCARE database (http://bioinformatics.psb.ugent.be/webtools/plantcare/html/, accessed on 26 September 2025).

### 4.5 RNA Extraction and RT‐qPCR

Total RNA was extracted from tissue samples using the HiPure Plant RNA Mini Kit (Magen) according to the manufacturer's instructions and stored at −80°C until use. First‐strand cDNA was synthesized using the HiScript III 1st Strand cDNA Synthesis Kit (Vazyme) with random primers, with 1 μg of total RNA added per 20 μL reaction volume. qPCR was performed using the SYBR Green qRT‐PCR kit (Vazyme) on an ABI 7900HT sequence detection system (Applied Biosystems QuantStudio 5) to measure the relative expression levels of the genes. At least three biological replicates and three technical replicates were included in all qPCR analyses. Each experiment was repeated at least three times. Relative gene expression was calculated using the 2^−∆∆*C*t^ method. The primers used in this study are listed in Table S5.

### 4.6 Plant Materials, Growth and Virus Inoculation

*Nicotiana benthamiana* seeds were germinated in a growth chamber at 25°C and 70% relative humidity under long‐day conditions (16 h light/8 h dark cycles). *Agrobacterium tumefaciens* GV3101 harbouring the PVX‐GFP (GFP‐labelled PVX) infectious clone was cultured. The resulting *Agrobacterium* cultures were pelleted and resuspended in an infiltration buffer (100 mM MES, pH 5.2, 10 mM MgCl_2_, 200 mM acetosyringone) to an OD_600_ of 0.5, followed by incubation for more than 2 h at 25°C. *Agrobacterium* harbouring PVX was then infiltrated into *N. benthamiana* leaves. The infiltrated plants were grown in a growth chamber maintained at 25°C, with a 14 h light/10 h dark cycle and 70% relative humidity.

### 4.7 Transgenic Overexpression and Gene Editing of *N. benthamiana*

Ten *N. benthamiana* seeds were washed with 75% ethanol for 1 min, then sterilized using 15% H_2_O_2_ for 15 min, after which they were washed three times using water for 3 min each time, spread on Murashige and Skoog (MS) medium (332 mg/L CaCl_2_, 170 mg/L KH_2_PO_4_, 1900 mg/L KNO_3_, 180 mg/L MgSO_4_, 0.83 mg/L KI and 8.6 mg/L ZnSO_4_) in an ultraclean bench, and cultured in a light incubator (GXZ‐1000, Ningbo Jiangnan Instrument Factory) at 28°C for about 15 days. *N. benthamiana* leaves were cut into 0.5 × 0.5 cm slices on an ultraclean table; the *N. benthamiana* leaves were transferred to the treated *Agrobacterium* spp. solution (OD_600_ about 0.5, CRISPR‐*NbDPB* plants were generated using a CRISPR/Cas9 vector carrying two gRNAs simultaneously targeting conserved regions of *NbDPB1* and *NbDPB2*, OE‐NbDPB use pRI101‐NbDPB‐GFP), and infiltrated for 5 min; the leaves were blotted dry on a sterile filter paper, and then inoculated into MS solid co‐culture medium (1/2 MS, 30 g/L sucrose, 8 g/L agar, pH 5.8) and put in the darkness at 22°C in a constant temperature box for 2 days. The leaves on the plate were transferred to induction medium (MS + 0.5 mg/L benzylaminopurine [BA], 30 g/L sucrose, 8 g/L agar, 500 mg/L cefotaxime, 100 mg/L kanamycin [Kan], pH 5.8). During placement, the wound sited were attached to the surface of the medium with the adaxial surface facing upwards, and after transferring, the leaves were sealed with a sealing film and put into a light incubator to be cultured for 15 days. After a transfer to the same medium at about 15 days, the leaves on the plate were then transferred to a differentiation medium (MS + 0.5 mg/L BA, 30 g/L sucrose, 8 g/L agar, pH 5.8) with wounded regions placed in contact with the medium surface and the adaxial side facing upward. When adventitious buds regenerated on the induction medium, these small shoots were transferred to rooting medium (MS + 0.1 mg/L NAA, 30 g/L sucrose, 8 g/L agar, 100 mg/L Kan, pH 5.8) and cultured under light conditions for 40 days to grow shoots and induce rooting.

### 4.8 Western Blot Assay

For total protein extraction, tissue samples were homogenized in a lysis buffer containing 60% SDS, 100 mM Tris–HCl (pH 8.8) and 2% β‐mercaptoethanol. Protein samples were analysed by SDS‐PAGE electrophoresis using precast 12% protein gel wells (HEPES‐Tris, Yeasen) and then transferred onto nitrocellulose membranes. The blots were incubated in blocking buffer (5% skimmed milk and 0.05% Tween 20 in 1 × Tris‐buffered saline [TBS]) for 60 min followed by detection with GFP antibody (TransGen Biotech), PVX CP antibody (HUABIO) and horseradish peroxidase (HRP)‐conjugated anti‐mouse or anti‐rabbit secondary antibodies (TransGen Biotech). Detection signals were visualized using an Amersham Imager 680 (GE Healthcare BioSciences).

### 4.9 RNA‐Seq Library Preparation for Transcriptome Sequencing

Total RNA of WT or *Cr‐nbdpb* was used as input material for the RNA sample preparations. Briefly, mRNA was purified from total RNA using poly‐T oligo‐attached magnetic beads. Fragmentation was carried out using divalent cations under elevated temperature in First Strand Synthesis Reaction Buffer (5×). First‐strand cDNA was synthesized using random hexamer primer and M‐MuLV reverse transcriptase (RNase H−). Second‐strand cDNA synthesis was subsequently performed using DNA polymerase I and RNase H. Remaining overhangs were converted into blunt ends via exonuclease/polymerase activities. After adenylation of 3′ ends of DNA fragments, Adaptor with hairpin loop structure was ligated to prepare for hybridization. In order to select cDNA fragments of preferentially 370–420 bp in length, the library fragments were purified with AMPure XP system. Then PCR was performed with Phusion High‐Fidelity DNA polymerase, Universal PCR primers and Index (X) Primer. At last, PCR products were purified (AMPure XP system) and library quality was assessed on the Agilent Bioanalyzer 2100 system. The experiment was conducted by Novogene company.

### 4.10 Bioinformatics Analysis of Differentially Expressed Genes (DEGs)

The Uniprot‐GOA database (http://www.ebi.ac.uk/GOA/, accessed on 15 November 2025) was used for Gene Ontology (GO) annotation. Proteins were classified into three categories based on GO annotation: biological processes, molecular functions and cellular components. The Kyoto Encyclopedia of Genes and Genomes (KEGG) database (http://www.genome.jp/kegg/genes.html, accessed on 15 November 2025) was used for KEGG annotation (Ogata et al. 1999). The pathways of the identified DEGs were annotated using KEGG, and the annotation results were mapped onto the KEGG pathway database using the KEGG Mapper online tool. Enrichment analysis of GO terms and KEGG pathways was performed using a two‐tailed Fisher's exact test. The *p*‐value was used to determine significant enrichment of GO terms and KEGG pathways, with *p* < 0.05 considered statistically significant.

### 4.11 ChIP‐Seq Analysis

ChIP experiments were conducted as previously reported (Lin et al. 2022). Briefly, 3 g of leaves from GFP‐positive *NbDPB‐OE N. benthamiana* plants selected from independent overexpression lines were pooled and crosslinked with 1% formaldehyde, and the reaction was quenched with 137.5 mM glycine. After nuclei isolation, chromatin was sonicated into 200–500 bp fragments using a Diagenode Bioruptor (high setting, 16 cycles of 30 s on/30 s off). The chromatin was then incubated with GFP antibodies (Abcam) for 8–12 h. Subsequently, the recovered DNA was purified and used as a template for ChIP‐Seq and ChIP‐qPCR assays. Two biological replicates were performed for each assay. Primers used in the experiments are listed and described in Table S5.

For ChIP‐Seq assays, recovered DNA was used for library construction with the Scale ssDNA‐seq Lib Prep Kit (ABclonal). High‐throughput sequencing was performed on the Illumina NovaSeq 6000 PE150 platform by LC‐Bio Technology (Hangzhou, China). Clean reads were mapped to the reference genome using Bowtie2 (v. 2.2.5) with default parameters. Reads with low mapping quality or multiple genomic alignments were removed using SAMTOOLS (v. 1.13). Enriched peaks were called using MACS2 (v. 2.2.6) for NbDPB occupancy with the command ‘callpeak ‐f BAMPE ‐B ‐q 0.05 ‐g 40949933’. Peaks were annotated using HOMER (v. 4.9.1) with the ‘annotatePeaks.pl’ script. Genes whose transcription start site or gene body was located within 3 kb of NbDPB ChIP‐seq peaks identified by MACS2 (*q* < 0.05) were defined as NbDPB‐binding.

### 4.12 Dual Luciferase Assays

For the transcriptional activity assays, the full‐length *NbDPB* gene was cloned into the pGWB505‐35S‐GFP vector as an effector, while the pGWB505‐35S‐GFP vector served as the negative control. The promoter of *NbMYB* was used to drive the firefly luciferase gene (*LUC*) as a reporter, and the Renilla luciferase (*REN*) gene controlled by a portion of the NbMYB promoter, was used as the reference. *A. tumefaciens* GV3101 strains harbouring combinations of effectors and reporters were infiltrated into *N. benthamiana* leaves and incubated for 48 h. Dual‐luciferase assays were performed using the Luciferase Reporter Assay System (Promega) according to the manufacturer's instructions. Relative luciferase activity was calculated using LUC/REN ratios. Each assay was conducted with three biological replicates, yielding consistent results. The primers used for these constructs are listed in Table S5.

### 4.13 VIGS

To silence *NbMYB* in *N. benthamiana*, a 337 bp fragment representing part of its sequence was inserted into the TRV2 vector (Liu et al. 2002). *Agrobacterium* cultures carrying pTRV1 and pTRV2 (referred to as TRV:00), as well as pTRV1 and pTRV2:*NbMYB* (TRV:*NbMYB*), were infiltrated into the leaves of *N. benthamiana* plants. The infiltrated plants were then grown in a climate‐controlled growth chamber at 25°C for 7 days, followed by a second inoculation with PVX via agroinfiltration and maintained at 25°C. Plants inoculated with TRV:00 served as controls.

## Author Contributions

**Jingjing Shi:** methodology, data curation, writing – original draft, formal analysis, investigation. **Lingyun Lei:** methodology. **Yumei Zhao:** methodology, investigation. **Jianping Chen:** funding acquisition. **Jian Yang:** writing – review and editing, funding acquisition, conceptualization. **Jiaqian Liu:** conceptualization. **Wenhao Wang:** methodology. **Peng Liu:** conceptualization. **Yaoyao Jiang:** methodology. **Bin Yong:** software, methodology. **Chunyan Qi:** investigation. **Kaili Zhong:** conceptualization, methodology, investigation, funding acquisition, writing – review and editing, project administration. **Tianye Zhang:** conceptualization. **Tianbo Liu:** funding acquisition, project administration.

## Funding

This work was supported by the National Key Research and Development Program of China (2023YFD1400300), Natural Science Foundation of Ningbo Municipality (2023J382) and China Agriculture Research System of MOF and MARA (CARS‐03).

## Conflicts of Interest

The authors declare no conflicts of interest.

## Supporting information


**Figure S1:** Identification and sequence logos of five conserved motifs in DP proteins. Sequence logos of the five conserved motifs identified in DP proteins from *Arabidopsis thaliana* , *Nicotiana benthamiana* and *Oryza sativa* generated using MEME Suite 5.5.2. The height of each amino acid letter at a given position represents the relative frequency (conservation) of that residue, with the overall height of the stack indicating the sequence conservation at that position (measured in bits). Motifs 1–5 are numbered sequentially, and the conserved residues are colour‐coded by amino acid property (hydrophobic, polar, charged, etc.).

**Figure S2:** Validation of *Cr‐nbdpb* mutants and *NbDPB* overexpression lines. (A) Sequence alignment of the target regions in wild‐type *Nicotiana benthamiana NbDPB1*/*NbDPB2* and CRISPR/Cas9‐edited *Cr‐nbdpb* mutant lines. The positions of gRNA1, gRNA2 and PAM sequences are indicated. Insertions and deletions generated in independent mutant lines are shown by sequence alignment. (B) Sanger sequencing chromatograms confirming mutations in representative *Cr‐nbdpb* lines. Sequence deletions around the CRISPR target sites were detected in all edited lines. (C) Immunoblot analysis of transiently expressed *NbDPB* overexpression constructs in *N. benthamiana*. Anti‐GFP antibody was used to detect NbDPB‐GFP fusion proteins. GFP alone was used as the control. The protein content in the different samples was then determined by Ponceau S staining.

**Figure S3:** Functional enrichment analyses of transcriptomic and chromatin immunoprecipitation (ChIP)‐seq datasets associated with NbDPB. (A) Gene Ontology enrichment analysis of differentially expressed genes identified from the transcriptome comparison between wild‐type (WT) and *Crnbdpb* plants. (B) KEGG pathway enrichment analysis of differentially expressed genes identified from the transcriptome comparison between WT and *Crnbdpb* plants. (C) GO enrichment analysis of genes associated with NbDPB binding peaks identified by ChIP‐seq in NbDPB‐OE plants. (D) KEGG pathway enrichment analysis of genes associated with NbDPB binding peaks identified by ChIP‐seq in OE‐DPB plants. Enriched GO terms and KEGG pathways are ranked according to their significance levels, and the size and colour of each dot represent the gene number and enrichment significance, respectively.

**Figure S4:** Phylogenetic analysis of GARP and bHLH transcription factor family in *Arabidopsisthaliana*. (A) Phylogenetic tree of *Arabidopsis* GARP family proteins and NbMYB, constructed using the neighbour‐joining method with 1000 bootstrap replicates. The tree is divided into two major clades, coloured by subfamily: red (ARR‐B subfamily) and blue (GOLDEN2‐LIKE subfamily). NbMYB is highlighted in red, clustering within the GOLDEN2‐LIKE subfamily. Bootstrap values (%) are indicated at the nodes. (B) Phylogenetic tree of *Arabidopsis* bHLH family proteins and NbUNE10, constructed using the maximum‐likelihood method with 1000 bootstrap replicates. The tree is divided into 12 distinct clades (I–XII), colour‐coded as indicated. NbUNE10 is highlighted in red, and bootstrap support values are represented by coloured dots at the nodes (scale bar: 0.1 amino acid substitutions per site).

**Figure S5:** Validation of *Cr‐nbe2f* mutant line. (A) Schematic representation of CRISPR/Cas9‐mediated knockdown of *NbE2F*. The PAM sequence (Protospacer Adjacent Motif) is the target DNA sequence to which Cas9 binds and cleaves. The red‐marked bases indicate the guide RNA (gRNA) sequence that recognizes the target genomic region. (B) Sequence alignment of the target regions in wild‐type *Nicotiana benthamiana NbE2F* and CRISPR/Cas9‐edited *Cr‐nbe2f* mutant line. The positions of gRNA1, gRNA2 and PAM sequences are indicated. Insertions and deletions generated in independent mutant lines are shown by sequence alignment. (C) Sanger sequencing chromatograms confirming mutation in *Cr‐nbe2f* line. Insertions in gRNA1 and deletions in gRNA2 were detected in the *Cr‐nbe2f* line.

**Figure S6:** Exogenous application of jasmonic acid (JA) and salicylic acid (SA) enhances resistance to potato virus X (PVX) infection in TRV:00 plants. (A) Systemic mosaic symptoms on the PVX‐inoculated TRV:00, TRV:00+JA and TRV:00+SA plants. Photographs were taken at 4 and 6 days post‐inoculation (dpi). Bar = 5 cm. (B) Western blot assay for PVX coat protein (CP) accumulation in PVX‐inoculated TRV:00, TRV:00+JA and TRV:00+SA plant leaves. The leaves' protein was taken at 4 and 6 dpi. The protein content in the different samples was then determined by Ponceau S staining. The numbers below the bands mean the intensity ratio of the three biological replicate bands that were calculated by ImageJ software. There were three biological replicates. (C) Relative expression levels of *NbMYB* in the TRV:00, TRV:*NbMYB‐1*, TRV:*NbMYB*‐2 and TRV:*NbMYB*‐3 plants was determined by reverse transcription‐quantitative PCR. Data presented are the mean ± SD of three biological samples per treatment. Significant differences between treatments were determined using one‐way ANOVA, **p < 0.01.

**Table S1:** Downregulated genes in *Cr‐nbdpb* versus wild‐type (WT).

**Table S2:** NbDPB‐binding genes identified by chromatin immunoprecipitation (ChIP)‐seq.

**Table S3:** Genes identified by Intersection analysis of transcriptomic data and chromatin immunoprecipitation (ChIP)‐seq data.

**Table S4:** All known motifs of binding peaks for 31 overlapping genes.

**Table S5:** Primers used in this study.

## Data Availability

The data that supports the findings of this study are available in the Figures [Link], [Link] and Tables [Link], [Link] of this article.
