# Supplementary figures and images for: The Transcription Factor DPB Confers Antiviral Defence Against Potato Virus X by Modulating MYB‐Dependent Signalling
Source: Mol Plant Pathol. 2026 Jul 12;27(7):e70319. doi: 10.1111/mpp.70319 (PMC13357984; doi:10.1111/mpp.70319)

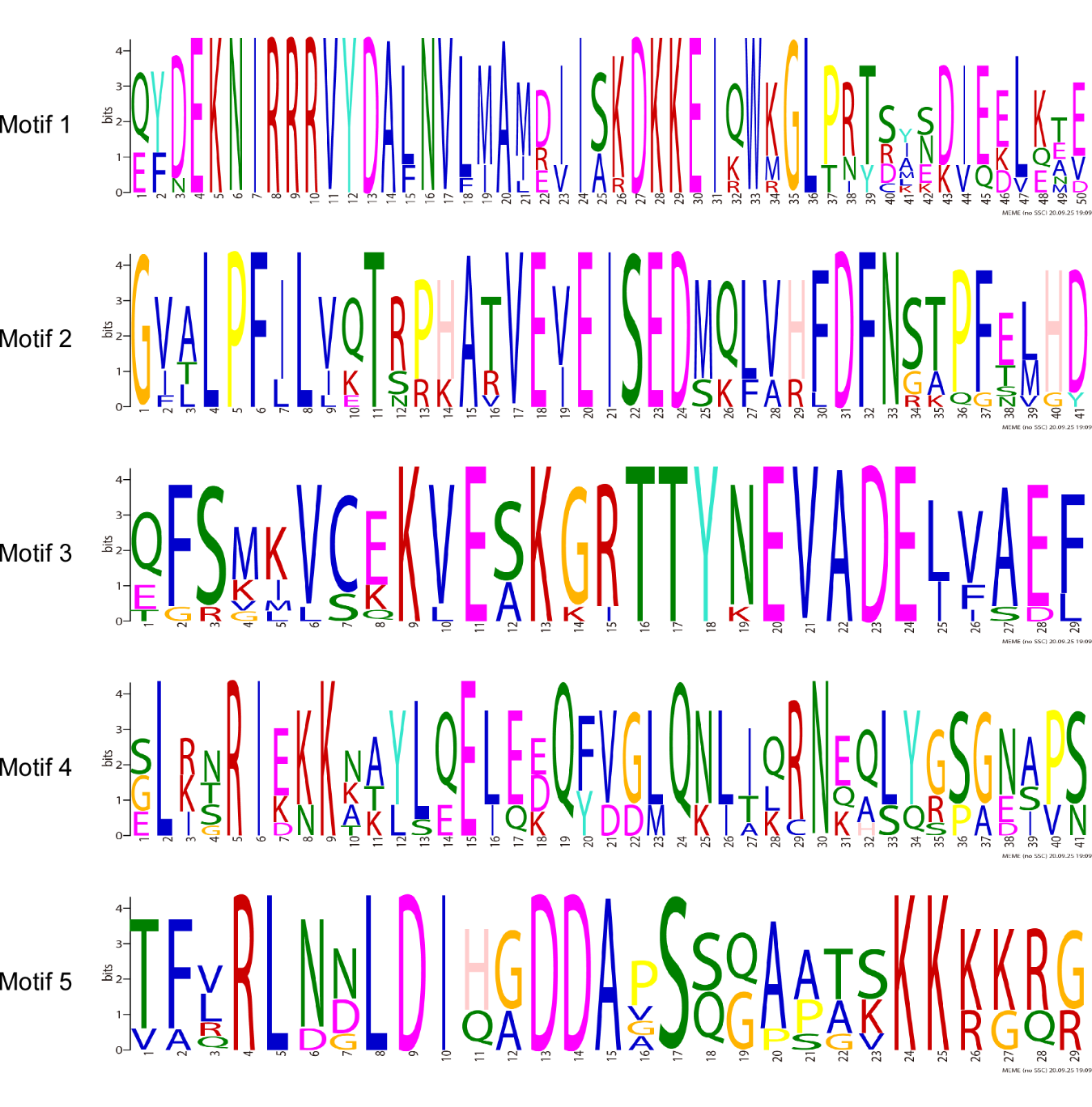

Supplement: Supplementary file 1 — Figure S1: Identification and sequence logos of five conserved motifs in DP proteins. Sequence logos of the five conserved motifs identified in DP proteins from Arabidopsis thaliana , Nicotiana benthamiana and Oryza sativa generated using MEME Suite 5.5.2. The height of each amino acid letter at a given position represents the relative frequency (conservation) of that residue, with the overall height of the stack indicating the sequence conservation at that position (measured in bits). Motifs 1–5 are numbered sequentially, and the conserved residues are colour‐coded by amino acid property (hydrophobic, polar, charged, etc.). [file MPP-27-e70319-s011.docx]

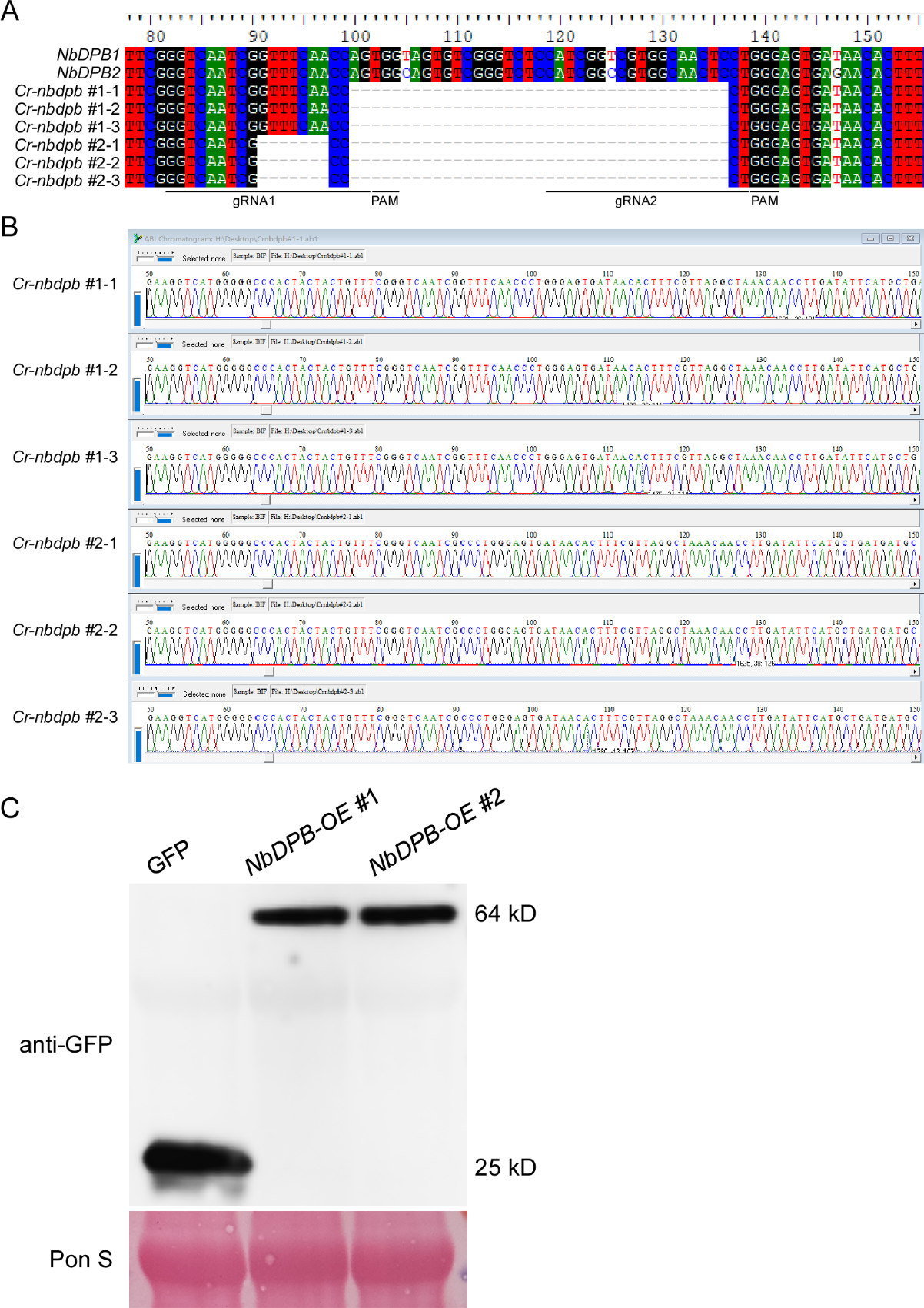

Supplement: Supplementary file 2 — Figure S2: Validation of Cr‐nbdpb mutants and NbDPB overexpression lines. (A) Sequence alignment of the target regions in wild‐type Nicotiana benthamiana NbDPB1/NbDPB2 and CRISPR/Cas9‐edited Cr‐nbdpb mutant lines. The positions of gRNA1, gRNA2 and PAM sequences are indicated. Insertions and deletions generated in independent mutant lines are shown by sequence alignment. (B) Sanger sequencing chromatograms confirming mutations in representative Cr‐nbdpb lines. Sequence deletions around the CRISPR target sites were detected in all edited lines. (C) Immunoblot analysis of transiently expressed NbDPB overexpression constructs in N. benthamiana. Anti‐GFP antibody was used to detect NbDPB‐GFP fusion proteins. GFP alone was used as the control. The protein content in the different samples was then determined by Ponceau S staining. [file MPP-27-e70319-s003.docx]

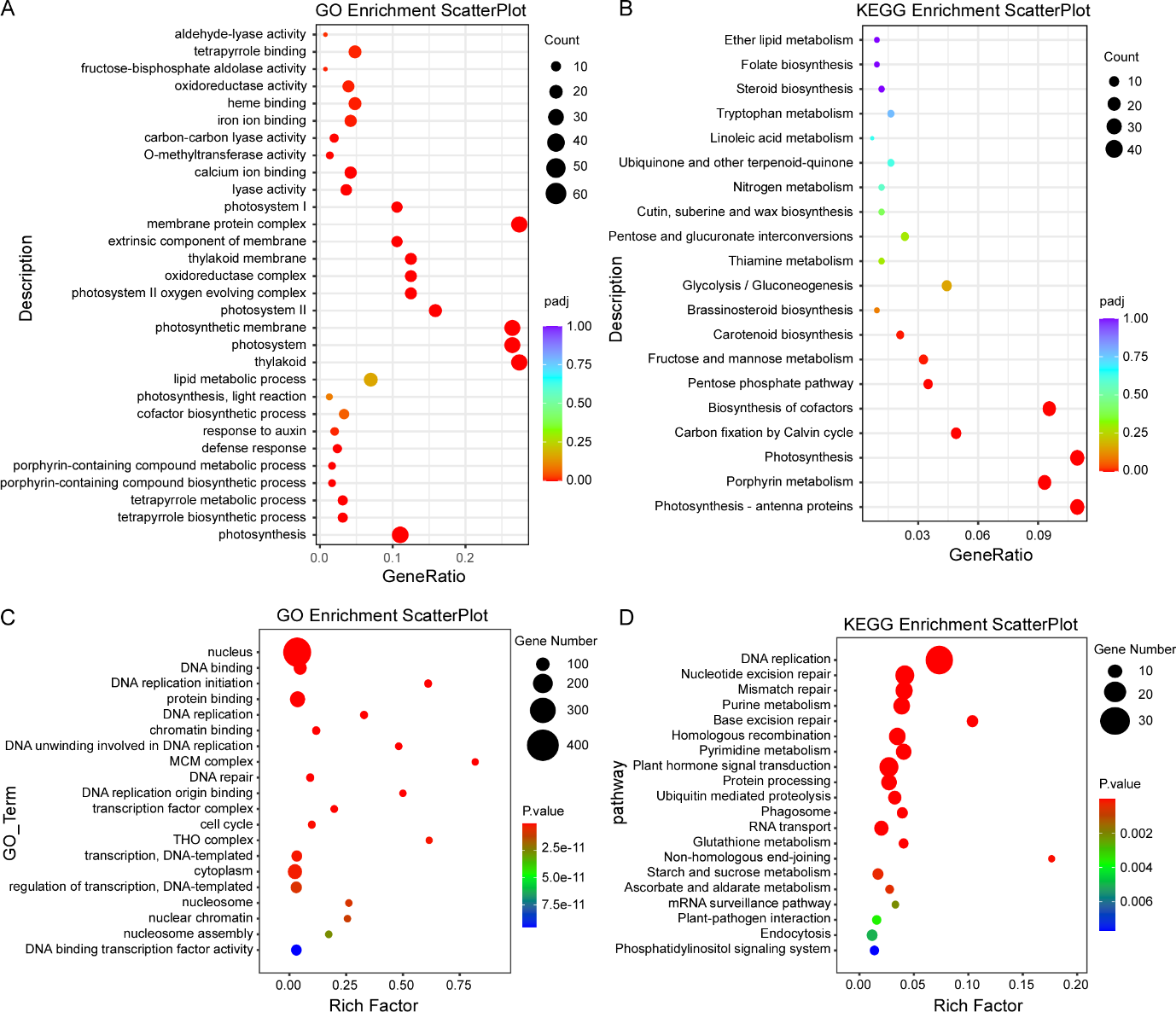

Supplement: Supplementary file 3 — Figure S3: Functional enrichment analyses of transcriptomic and chromatin immunoprecipitation (ChIP)‐seq datasets associated with NbDPB. (A) Gene Ontology enrichment analysis of differentially expressed genes identified from the transcriptome comparison between wild‐type (WT) and Crnbdpb plants. (B) KEGG pathway enrichment analysis of differentially expressed genes identified from the transcriptome comparison between WT and Crnbdpb plants. (C) GO enrichment analysis of genes associated with NbDPB binding peaks identified by ChIP‐seq in NbDPB‐OE plants. (D) KEGG pathway enrichment analysis of genes associated with NbDPB binding peaks identified by ChIP‐seq in OE‐DPB plants. Enriched GO terms and KEGG pathways are ranked according to their significance levels, and the size and colour of each dot represent the gene number and enrichment significance, respectively. [file MPP-27-e70319-s010.docx]

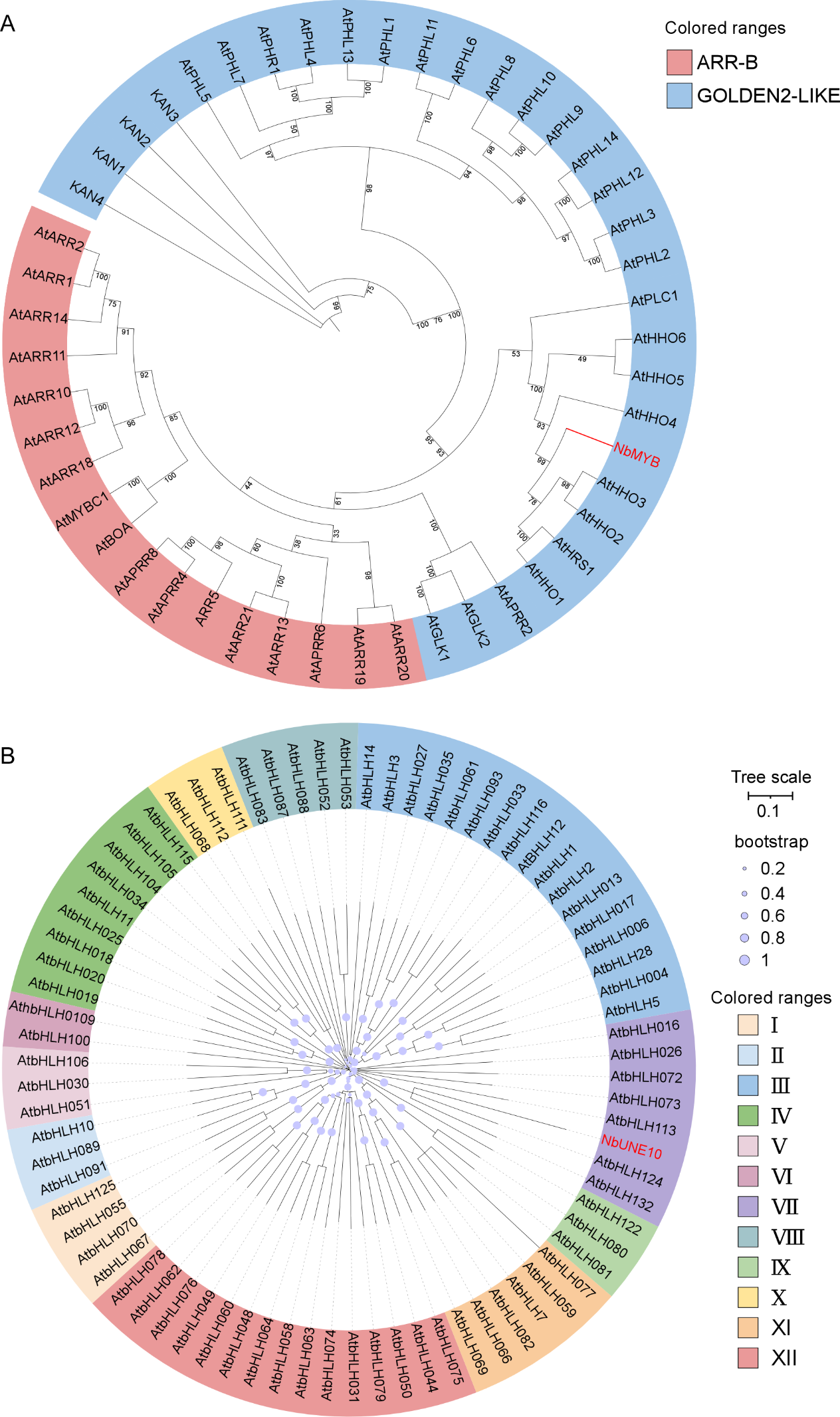

Supplement: Supplementary file 4 — Figure S4: Phylogenetic analysis of GARP and bHLH transcription factor family in Arabidopsisthaliana. (A) Phylogenetic tree of Arabidopsis GARP family proteins and NbMYB, constructed using the neighbour‐joining method with 1000 bootstrap replicates. The tree is divided into two major clades, coloured by subfamily: red (ARR‐B subfamily) and blue (GOLDEN2‐LIKE subfamily). NbMYB is highlighted in red, clustering within the GOLDEN2‐LIKE subfamily. Bootstrap values (%) are indicated at the nodes. (B) Phylogenetic tree of Arabidopsis bHLH family proteins and NbUNE10, constructed using the maximum‐likelihood method with 1000 bootstrap replicates. The tree is divided into 12 distinct clades (I–XII), colour‐coded as indicated. NbUNE10 is highlighted in red, and bootstrap support values are represented by coloured dots at the nodes (scale bar: 0.1 amino acid substitutions per site). [file MPP-27-e70319-s004.docx]

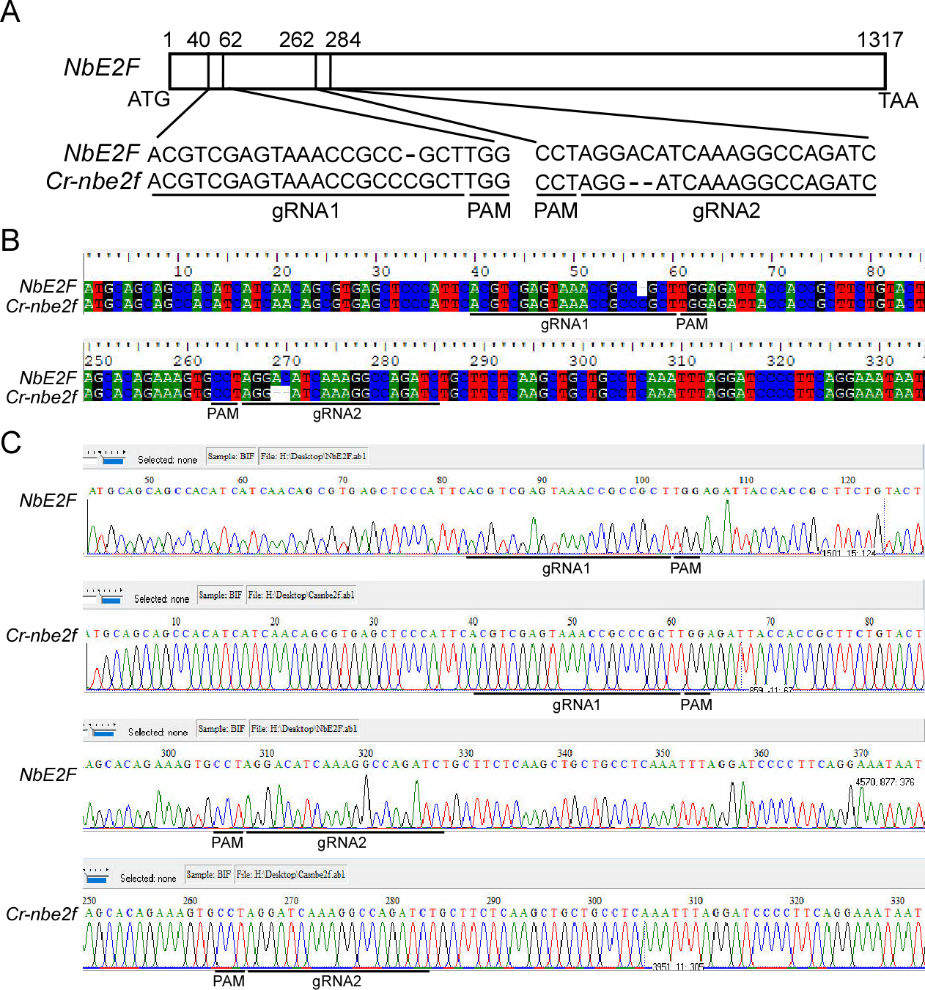

Supplement: Supplementary file 5 — Figure S5: Validation of Cr‐nbe2f mutant line. (A) Schematic representation of CRISPR/Cas9‐mediated knockdown of NbE2F. The PAM sequence (Protospacer Adjacent Motif) is the target DNA sequence to which Cas9 binds and cleaves. The red‐marked bases indicate the guide RNA (gRNA) sequence that recognizes the target genomic region. (B) Sequence alignment of the target regions in wild‐type Nicotiana benthamiana NbE2F and CRISPR/Cas9‐edited Cr‐nbe2f mutant line. The positions of gRNA1, gRNA2 and PAM sequences are indicated. Insertions and deletions generated in independent mutant lines are shown by sequence alignment. (C) Sanger sequencing chromatograms confirming mutation in Cr‐nbe2f line. Insertions in gRNA1 and deletions in gRNA2 were detected in the Cr‐nbe2f line. [file MPP-27-e70319-s007.docx]

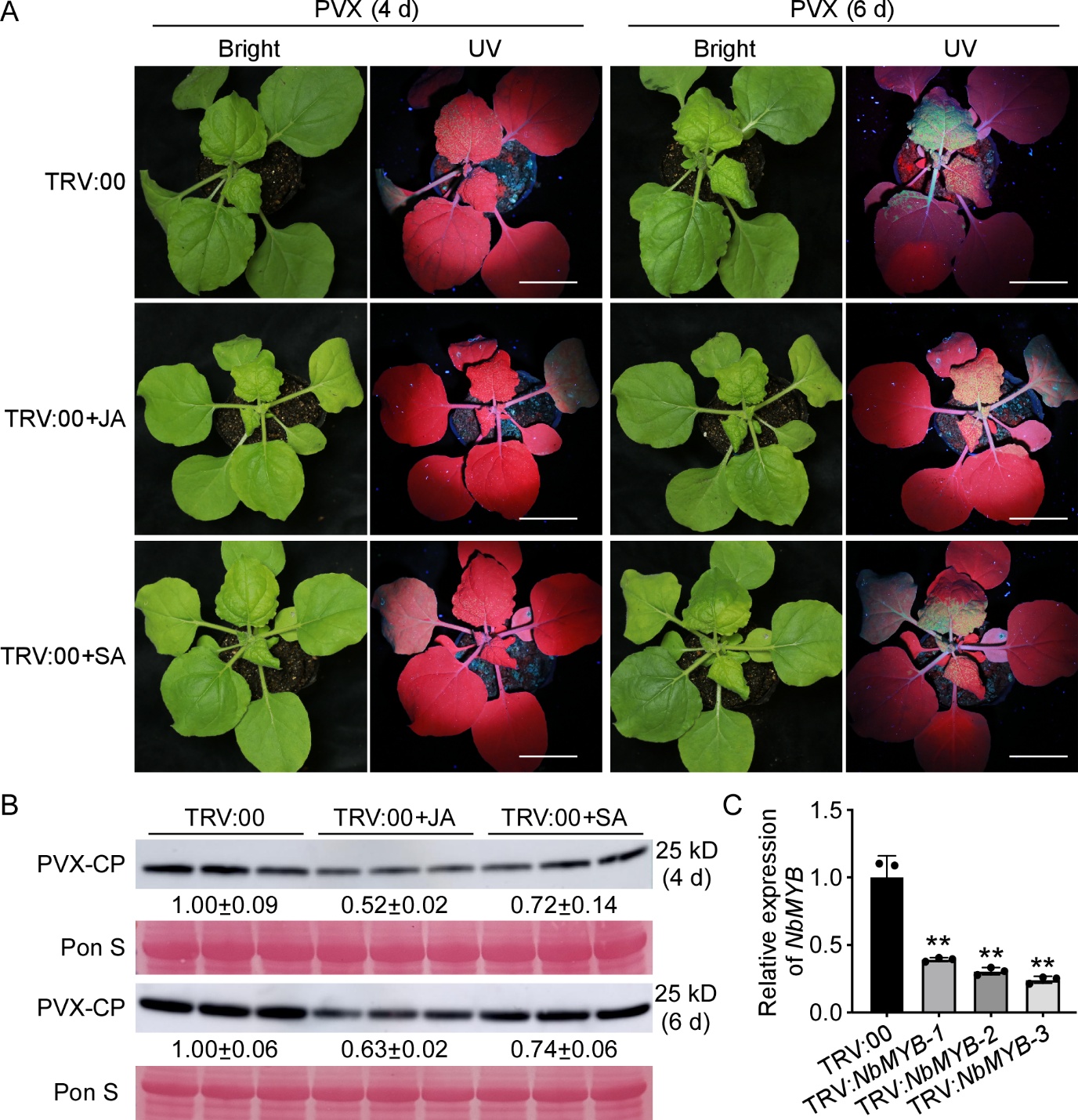

Supplement: Supplementary file 6 — Figure S6: Exogenous application of jasmonic acid (JA) and salicylic acid (SA) enhances resistance to potato virus X (PVX) infection in TRV:00 plants. (A) Systemic mosaic symptoms on the PVX‐inoculated TRV:00, TRV:00+JA and TRV:00+SA plants. Photographs were taken at 4 and 6 days post‐inoculation (dpi). Bar = 5 cm. (B) Western blot assay for PVX coat protein (CP) accumulation in PVX‐inoculated TRV:00, TRV:00+JA and TRV:00+SA plant leaves. The leaves' protein was taken at 4 and 6 dpi. The protein content in the different samples was then determined by Ponceau S staining. The numbers below the bands mean the intensity ratio of the three biological replicate bands that were calculated by ImageJ software. There were three biological replicates. (C) Relative expression levels of NbMYB in the TRV:00, TRV:NbMYB‐1, TRV:NbMYB‐2 and TRV:NbMYB‐3 plants was determined by reverse transcription‐quantitative PCR. Data presented are the mean ± SD of three biological samples per treatment. Significant differences between treatments were determined using one‐way ANOVA, **p < 0.01. [file MPP-27-e70319-s001.docx]
