# Supplementary material for: The Transcription Factor DPB Confers Antiviral Defence Against Potato Virus X by Modulating MYB‐Dependent Signalling
Source: Mol Plant Pathol. 2026 Jul 12;27(7):e70319. doi: 10.1111/mpp.70319 (PMC13357984; doi:10.1111/mpp.70319)
Supplement: Supplementary file 10 — Table S4: All known motifs of binding peaks for 31 overlapping genes. [file MPP-27-e70319-s005.docx]

Table S4 All known motifs of binding peaks for 31 overlapping genes

| gene ID | Sequence | Motif Name | |
| --- | --- | --- | --- |
| Niben261Chr01g0054001 | TTTCACATGTGGAAC | bZIP3(bZIP)/col-bZIP3-DAP-Seq(GSE60143)/Homer |  |
| Niben261Chr01g0633003 | CTCCGCCTTTAT | AT1G71450(AP2EREBP)/col-AT1G71450-DAP-Seq(GSE60143)/Homer |  |
| Niben261Chr02g0594003 | TTCCACGTAA | bZIP16(bZIP)/colamp-bZIP16-DAP-Seq(GSE60143)/Homer |  |
| Niben261Chr02g0981002 | TTACCTCTCTTTTTC | GAGA-repeat/SacCer-Promoters/Homer |  |
| Niben261Chr03g1213007 | CAAACAAATG | HuR/HEK293-HuR-CLIP-Seq(GSE87887)/Homer |  |
| Niben261Chr05g0663002 | AATAAAAGCA | dof45(C2C2dof)/col-dof45-DAP-Seq(GSE60143)/Homer |  |
| Niben261Chr05g0934012 | GAAAGTGAAATG | PRDM1(Zf)/Hela-PRDM1-ChIP-Seq(GSE31477)/Homer |  |
| Niben261Chr05g0940001 | TTGGGCGCGATC | E2F3(E2F)/MEF-E2F3-ChIP-Seq(GSE71376)/Homer |  |
| Niben261Chr06g0801016 | TACCCGTGACGTGGC | bZIP48(bZIP)/colamp-bZIP48-DAP-Seq(GSE60143)/Homer |  |
| Niben261Chr06g1111014 | AAGATGAAGGGAGAA | GAGA-repeat/SacCer-Promoters/Homer |  |
| Niben261Chr08g0046009 | ATTGTCACGTCATCA | GBF5(bZIP)/colamp-GBF5-DAP-Seq(GSE60143)/Homer |  |
| Niben261Chr08g0185006 | CCAAACACGTGGATC | AREB3(bZIP)/col-AREB3-DAP-Seq(GSE60143)/Homer |  |
| Niben261Chr09g1252003 | TGGAGGGAGA | SeqBias: GA-repeat |  |
| Niben261Chr10g0473012 | GTTACCCGGGCCCAC | TCP16(TCP)/colamp-TCP16-DAP-Seq(GSE60143)/Homer |  |
| Niben261Chr10g1181001 | GTGGGCCCAATGGAG | TCP16(TCP)/colamp-TCP16-DAP-Seq(GSE60143)/Homer |  |
| Niben261Chr10g1199005 | TGGTGGGTCCAGGTC | At1g69690(TCP)/colamp-At1g69690-DAP-Seq(GSE60143)/Homer |  |
| Niben261Chr10g1216004 | TCAACTGTTT | BMYB(HTH)/Hela-BMYB-ChIP-Seq(GSE27030)/Homer |  |
| Niben261Chr11g1226004 | TTAACCGTAC | MYB77(MYB)/col-MYB77-DAP-Seq(GSE60143)/Homer |  |
| Niben261Chr12g0216014 | GAGCCGTTGA | MYB77(MYB)/col-MYB77-DAP-Seq(GSE60143)/Homer |  |
| Niben261Chr12g0227008 | TAGCCGTCGCTG | RAP26(AP2EREBP)/colamp-RAP26-DAP-Seq(GSE60143)/Homer |  |
| Niben261Chr12g0265014 | CCGCCGTGACAA | RAP26(AP2EREBP)/colamp-RAP26-DAP-Seq(GSE60143)/Homer |  |
| Niben261Chr12g1051002 | GGCGGCAAAA | DEL2(E2FDP)/col-DEL2-DAP-Seq(GSE60143)/Homer |  |
| Niben261Chr12g1301004 | TTTTGTGTTT | HuR/HEK293-HuR-CLIP-Seq(GSE87887)/Homer |  |
| Niben261Chr13g0349001 | TTACCGACTTTA | DEAR2(AP2EREBP)/colamp-DEAR2-DAP-Seq(GSE60143)/Homer |  |
| Niben261Chr13g1100004 | CCCCAACTTTTT | At5g62940(C2C2dof)/col-At5g62940-DAP-Seq(GSE60143)/Homer |  |
| Niben261Chr16g0319003 | TGTCGGTA | AT1G12630(AP2EREBP)/colamp-AT1G12630-DAP-Seq(GSE60143)/Homer |  |
| Niben261Chr16g0875002 | GTGTGGGTCCTATGG | At1g69690(TCP)/colamp-At1g69690-DAP-Seq(GSE60143)/Homer |  |
| Niben261Chr17g1369010 | CACTCCCGCC | SeqBias: CG bias |  |
| Niben261Chr17g1537013 | GGTATCCGTTTTAGA | MYB73(MYB)/col-MYB73-DAP-Seq(GSE60143)/Homer |  |
| Niben261Chr18g1461007 | ACACTTTATG | AT2G28810(C2C2dof)/colamp-AT2G28810-DAP-Seq(GSE60143)/Homer |  |
| Niben261Chr19g0530002 | AATAAAGGTC | AT1G47655(C2C2dof)/colamp-AT1G47655-DAP-Seq(GSE60143)/Homer |  |
